# To electrify bilingualism: Electrophysiological insights into bilingual metaphor comprehension

**DOI:** 10.1371/journal.pone.0175578

**Published:** 2017-04-17

**Authors:** Katarzyna Jankowiak, Karolina Rataj, Ryszard Naskręcki

**Affiliations:** 1 Faculty of English, Adam Mickiewicz University in Poznań, Poznań, Poland; 2 NanoBioMedical Centre, Adam Mickiewicz University in Poznań, Poznań, Poland; 3 Laboratory of Vision Science and Optometry, Faculty of Physics, Adam Mickiewicz University in Poznań, Poznań, Poland; University of Zurich, SWITZERLAND

## Abstract

Though metaphoric language comprehension has previously been investigated with event-related potentials, little attention has been devoted to extending this research from the monolingual to the bilingual context. In the current study, late proficient unbalanced Polish (L1)–English (L2) bilinguals performed a semantic decision task to novel metaphoric, conventional metaphoric, literal, and anomalous word pairs presented in L1 and L2. The results showed more pronounced P200 amplitudes to L2 than L1, which can be accounted for by differences in the subjective frequency of the native and non-native lexical items. Within the early N400 time window (300–400 ms), L2 word dyads evoked delayed and attenuated amplitudes relative to L1 word pairs, possibly indicating extended lexical search during foreign language processing, and weaker semantic interconnectivity for L2 compared to L1 words within the memory system. The effect of utterance type was observed within the late N400 time window (400–500 ms), with smallest amplitudes evoked by literal, followed by conventional metaphoric, novel metaphoric, and anomalous word dyads. Such findings are interpreted as reflecting more resource intensive cognitive mechanisms governing novel compared to conventional metaphor comprehension in both the native and non-native language. Within the late positivity time window (500–800 ms), Polish novel metaphors evoked reduced amplitudes relative to literal utterances. In English, on the other hand, this effect was observed for both novel and conventional metaphoric word dyads. This finding might indicate continued effort in information retrieval or access to the non-literal route during novel metaphor comprehension in L1, and during novel and conventional metaphor comprehension in L2. Altogether, the present results point to decreased automaticity of cognitive mechanisms engaged in non-native and non-dominant language processing, and suggest a decreased sensitivity to the levels of conventionality of metaphoric meanings in late proficient unbalanced bilingual speakers.

## Introduction

Metaphors are defined as expressions whose intended meanings do not correspond to their literal interpretation [1], which has raised the question of how speakers are able to arrive at nonliteral meanings. Behavioral and electrophysiological research into figurative language processing has repeatedly shown that metaphor processing is modulated by the level of conventionality of a presented metaphor [2–12]. Although previous research evinces distinct resource intensity of cognitive mechanisms governing novel and familiar metaphor comprehension in monolinguals, thus far only little attention has been devoted to examining how bilingual speakers process nonliteral meanings [13–16]. To the best of our knowledge, no behavioral or event-related potential (ERP) studies have compared novel and conventional metaphor processing in L1 and L2. In the current study, we aimed to test it by investigating the N400 and late positivity patterns while participants performed a semantic decision task in response to novel and conventional metaphoric, as well as literal and anomalous word dyads in L1 and L2. Below we discuss the main findings regarding ERP studies on metaphor comprehension in monolinguals and their implications for studies on bilinguals.

ERP studies investigating metaphoric and literal language processing in the monolingual context have often examined the N400 component [17,18]. The N400 is a negative-going wave that peaks in amplitude at around 400 ms after stimulus onset, and has a centro-parietal scalp distribution [19–21]. Not only has the N400 response been linked to lexical access and semantic information retrieval, but it has also been found to be modulated by the preceding contextual information [22–24]. Therefore, the component is frequently referred to as an index of lexico-semantic memory, and more pronounced N400 amplitudes reflect additional long-term memory processes engaged in information retrieval [25]. A larger N400 response to metaphoric compared to literal utterances frequently observed in previous studies might therefore index the increasing difficulty of mapping construction, which refers to the creation of relational correspondences between two concepts [17,18].

In monolingual research on figurative meaning processing, a modulation of the N400 amplitudes by the degree of metaphor conventionality has been reported in several studies [26–29]. Arzouan et al. [18] found a linear effect with smallest N400 amplitudes for literal (e.g., *burning fire*), followed by conventional metaphoric (e.g., *transparent intention*), novel metaphoric (e.g., *conscience storm*), and anomalous utterances (e.g., *indirect blanket*) in both a semantic decision task and a self-paced reading task. Such findings were interpreted as indicative of increasing effort in establishing mappings between concepts from literal, to conventional metaphoric, novel metaphoric, and anomalous utterances. Similar findings were shown by Lai et al. [29], who additionally divided the N400 time window into the early (320–440 ms) and late (440–560 ms) N400 effect. Within the early N400 time frame, the authors observed a larger N400 response to novel metaphors, conventional metaphors, and anomalous sentences compared to literal utterances. Within the late N400 time window, on the other hand, more pronounced N400 amplitudes were found to novel metaphors and anomalous utterances, yet not to conventional metaphors, for which the N400 amplitudes converged with those for literal utterances. Such findings point to increased cognitive demands in conventional compared to literal, and novel compared to conventional metaphor processing.

More recently, Goldstein et al. [30] observed the N400 conventionalization effect, in which novel metaphors which had previously been explained by participants in the exposure phase elicited a smaller N400 response in the subsequent test phase compared to unexplained utterances. In contrast, conventional metaphors which had been explained in the exposure phase evoked larger N400 amplitudes in the test phase relative to unexplained sentences. Such results seem to be in line with the Career of Metaphor Model [5], which postulates that metaphoric language comprehension is modulated by the degree of conventionality. According to this model, novel metaphors are comprehended by means of comparison. Namely, in a novel metaphor such as *theories are fathers*, a metaphoric target concept (*theories*) is structurally aligned with a literal base concept (*fathers*). Conventional metaphors, on the other hand, are preferentially analyzed as categorization, which refers to perceiving the metaphoric target concept as a member of a superordinate category specified by the literal base term. Thus, while novel metaphors require processes engaged in sense creation, conventional expressions are argued to involve sense retrieval, and are thus easier to comprehend, as revealed by attenuated N400 amplitudes for conventional compared to novel metaphors [18,29]. The findings observed by Goldstein et al. therefore demonstrate that explaining conventional metaphors might have activated comparison processes, resulting in additional activation of semantic information, and consequently a larger N400 amplitude. Novel metaphors, in contrast, may have undergone the process of conventionalization, which resulted in a reduced N400 response.

The tenets of the Career of Metaphor Model were also supported by Lai and Curran [31], who employed the priming paradigm, in which novel and conventional metaphoric sentences were primed by either sentence-primes (*A is B*) or simile-primes (*A is like B*). The findings showed that simile-primes, which initiate comparison processes, were effective in priming both novel and conventional metaphors, as reflected in reduced N400 amplitudes. In contrast, sentence-primes primed only conventional metaphors, which indicates that comparison processes are necessary when interpreting novel metaphors, and facilitatory in conventional metaphor comprehension. Thus, although comparison processes might play a vital role in conceptual mapping, it is conventionality that modulates the difficulty of this mapping, as argued by the Career of Metaphor Model.

Few studies on metaphor comprehension have discussed the late positive complex (LPC), which is a positive-going wave peaking in amplitude at 500–900 ms after stimulus onset [32,33]. In addition to its sensitivity to syntactic violations [34–36], late positivity is also modulated by semantic expectancy, semantic incongruencies on the sentence and discourse levels, and conceptual complexity of sentences [17,37–40]. This suggests that the LPC also reflects cognitive mechanisms engaged in meaning construction and revision, with the robustness of the LPC effect indexing the effort involved in these operations, as well as additional working memory processes necessary during meaning integration [41–43].

In previous studies, metaphors have evoked both increased and reduced LPC amplitudes compared to literal utterances. De Grauwe et al. [1] observed more robust LPC amplitudes in response to familiar metaphors than both anomalous and literal sentences. Such an effect was interpreted as reflecting additional reanalysis processes needed to resolve a conflict between the literal and metaphoric interpretation. An increased LPC response to conventional metaphors relative to anomalous utterances was also observed by Arzouan et al. [18], who at the same time found reduced LPC amplitudes for novel metaphoric word dyads, which remains difficult to account for within the current functional interpretations of this component. They postulated that sustained negativity overlapped with the LPC effect, thus reducing its amplitude. This late negativity might manifest continued effort related to information retrieval or access to the non-literal route. Such inconclusive findings are likely to result from different criteria for cloze probability and levels of metaphor conventionality applied in these studies, and thus further research is necessary to elucidate the precise functional role of the LPC response in metaphoric meaning processing.

Thus far little attention has been devoted to studying how the bilingual brain computes metaphoric utterances, while extending electrophysiological research on metaphoric language comprehension from the monolingual to the bilingual context could contribute to explicating mechanisms engaged when processing semantically complex meanings. Previous electrophysiological studies have looked into various other aspects of semantic processing in bilingualism, such as semantic priming or the processing of semantically congruous as opposed to incongruous stimuli, and have frequently reported a delay in the N400 peak latency to L2 relative to L1 stimuli [44–48]. This effect was interpreted as reflecting extended lexical search and less automatic lexical access in L2 than L1. Such a delayed N400 response to the non-native language is in line with the temporal delay assumption postulated by the Bilingual Interactive Activation Plus Model (BIA+, [49]), which stipulates a delayed activation of semantic representations in L2 compared to L1 due to lower subjective frequency of the non-native tongue. This delay is argued to result from differences between native and non-native language dominance, which pertains to the frequency of use of a given language. Namely, for L2 non-dominant (unbalanced) bilinguals, who use their non-native tongue less frequently than the native language, L2 lexical items are of lower subjective frequency compared to L1 words, which results in extended lexical search when processing the non-native tongue, as might be indexed by delayed N400 amplitudes.

Several experiments have reported an attenuated N400 response to L2 compared to L1 materials [50–54], which might index decreased interconnectivity for L2 words within the semantic network compared to L1 items [51]. Such weaker interconnectivity might in turn evoke decreased activity in long-term memory when processing the non-native language. Consequently, this interpretation seems to be in accordance with the functional role of the N400 effect, which links the N400 response to memory operations involved in information retrieval [23,25].

The present study provides an extension of research on semantic processing in bilingualism by using semantically simple (literal) as well as complex (novel metaphoric) utterances. Our experiment employed a within-subject design, and thus the potential influence of any between-group individual differences was minimized. Consequently, the study provides a direct comparison of cognitive mechanisms engaged in native and non-native nonliteral language comprehension, which can further elucidate how language proficiency, language dominance, and semantic complexity interact with one another during bilingual language processing.

The main objective of the study was to examine brain responses to novel metaphoric, conventional metaphoric, literal, and anomalous word pairs in Polish (L1) and English (L2). Our first aim was to investigate whether metaphoric meanings would evoke more pronounced N400 amplitudes compared to literal word dyads in both languages. Such an effect has been previously observed in monolingual research and was interpreted as indicative of more resource intensive mappings required for metaphor comprehension when compared to literal utterance understanding [5,17,31]. Additionally, we wanted to examine whether conventionality would modulate the N400 effect in both languages. Two previous studies have reported a linear N400 effect in the monolingual context [18,29], and interpreted larger N400 amplitudes for novel than conventional metaphors as indicating an increased processing difficulty due to sense creation mechanisms that are required when arriving at novel meanings. Our second aim was to test whether English word dyads would elicit attenuated and delayed effects within the N400 time frame compared to Polish word pairs, as has been observed in previous research on semantic processing in bilinguals [44–47,50–53]. A delay in the N400 peak latency to English relative to Polish stimuli would suggest extended lexical search during L2 processing due to possible differences in the subjective frequency of L2 lexical items, and would provide support for the temporal delay assumption postulated by the BIA+ [49]. An attenuated N400 response to English compared to Polish materials would additionally point to weaker semantic interconnectivity for L2 relative to L1 words [51]. Our third aim was to examine whether cognitive mechanisms involved in meaning integration, as indexed by the LPC response, are modulated by language nativeness. Since increased LPC amplitudes have been shown to index secondary semantic integration in previous monolingual research [1,41–43], we expected that more pronounced LPC amplitudes in response to anomalous and metaphoric than literal word dyads would be observed. Furthermore, if conventionality modulates the LPC effect, as shown by Arzouan et al. [18], then novel metaphors should evoke smaller LPC amplitudes than conventional metaphoric word dyads, indicating continued effort related to information retrieval or access to the non-literal route. Finally, a comparable LPC effect in both languages would indicate similar sensitivity to different levels of conventionality of metaphoric utterances in both languages.

## Method

### Participants

The original sample included 28 participants. Three of them had to be excluded from the analyses due to low accuracy rates on literal trials in Polish and English (below 70%), and one due to low accuracy rates on anomalous word dyads in English (26%). Additionally, one participant had to be removed from further analyses owing to an elevated impedance of the electrodes during the EEG recording. This resulted in a final sample of 23 native speakers of Polish (16 women, 7 men), aged 21–25 (*M*_*age*_ = 22.83, *SD* = 1.03), who were 1 MA and 2 MA students of the Faculty of English at Adam Mickiewicz University in Poznań, and who participated in the experiment for course credits. Scores from an online Handedness Questionnaire (Cohen, 2008) based on the Edinburgh Inventory [55] ranged from 50 to 100 (*M* = 89.8%, *SD* = 12.38), indicating right-hand preference for all participants. Participants were late proficient unbalanced students of English as their second language (*M*_*AgeofAcquisition*_ = 9.61, *SD* = 3.19). Due to the fact that participants were MA students of English Studies, they were all highly proficient in English. The program of English Studies at the Faculty of English, Adam Mickiewicz University in Poznań offers an intensive English-only curriculum, and ascertains students’ L2 proficiency level every year by means of administering standardized language examinations, whose levels of difficulty correspond to those defined within the Common European Framework of Reference for Languages (CEF). Participants recruited for the present study had all passed their Practical English Language Exam, which was equivalent to Cambridge Proficiency Examination administered by Cambridge University, and the passing of which was analogous to obtaining C2 proficiency level within the CEF. Apart from English, they had been learning other foreign languages (*M*_*AgeofAcquisition*_ = 17.06, *SD* = 4.63), such as German (39% of participants), Spanish (22%), French (22%), Swedish (4%), Italian (4%), Russian (4%), and Afrikaans (4%). All of the participants had normal or corrected to normal vision, and they did not suffer from any language or neurological disorder.

### Materials

Materials used in the study consisted of 304 Polish and 304 English verb-noun word dyads: 76 novel metaphors (e.g., *to harvest courage*), 76 conventional metaphors (e.g., *to gather courage*), 76 literal expressions (e.g., *to experience courage*), and 76 anomalous utterances (e.g., *to move courage*) in each language. Selected stimuli are provided in Table 1. Each set shared the same critical word (a noun), which was preceded by different prime words (verbs), depending on the condition. The criteria under which the critical words were selected are provided in Table 2. Due to difficulty in selecting stimuli that follow such criteria, the critical items used in the experiment included 9 Polish-English cognate words (8 in the English set and 1 in the Polish set of materials), which however constituted only 6% of all critical words.

**Table 1:** Selected sets of experimental stimuli.

Novel metaphors	Conventional metaphors	Literal utterances	Anomalous utterances
*to harvest courage*	*to gather courage*	*to experience courage*	*to move courage*
*to offend a custom*	*to adopt a custom*	*to learn a custom*	*to water a custom*
*to smell excuses*	*to invent excuses*	*to believe excuses*	*to hit excuses*
*to bury liberty*	*to exercise liberty*	*to guarantee liberty*	*to reprint liberty*
*to breed rumors*	*to silence rumors*	*to deny rumors*	*to cry rumors*

**Table 2:** Polish and English critical words' characteristics, including frequency per million, number of syllables, and number of letters.

	Frequency per million	Number of syllables	Number of letters
Polish (L1) critical words	15-36/million *(M* = 23.53, *SD* = 5.91); National Corpus of Polish Language	2–3 syllables *(M* = 2.49, *SD* = 0.5)	4–13 letters *(M* = 7.29, *SD* = 1.88)
English (L2) critical words	14-37/million *(M* = 23.75, *SD* = 6.41); Corpus of Contemporary American English, COCA	2–3 syllables *(M* = 2.51, *SD* = 0.5)	5–13 letters *(M* = 7.83, *SD* = 1.68)

In order to ensure that the stimuli were adequate representatives of the categories ascribed to them, they were pretested using web-based Likert-type surveys. Critical words were pretested in order to ensure that they were all abstract words. Word dyads were pretested in four normative studies: the cloze probability test, as well as meaningfulness, familiarity, and metaphoricity ratings. While Polish experimental stimuli were rated by native speakers of Polish, English materials were assessed by English native speakers. Raters whose scores were more than 3 SDs from the mean were excluded from final analyses. Table 3 provides the number of raters included in the analyses together with their demographic data. The raters who took part in these pretests did not participate in the ERP experiment.

**Table 3:** Demographic information concerning the participants of the five normative studies in each language, including the number of raters, their gender, and mean age.

Survey type	Language	Number of raters included in the analyses	Mean age
Critical words’ concreteness	Polish (L1)	N = 33 (23 women, 10 men)	*M*_*age*_ = 22.12, *SD* = 3.52
	English (L2)	N = 34 (19 women, 15 men)	*M*_*age*_ = 24.53, *SD* = 5.43
Cloze probability	Polish (L1)	N = 140 (112 women, 28 men)	*M*_*age*_ = 21.07, *SD* = 2.62
	English (L2)	N = 140 (65 women, 75 men)	*M*_*age*_ = 22.76, *SD* = 4.86
Meaningfulness	Polish (L1)	N = 137 (99 women, 38 men)	*M*_*age*_ = 21.52, *SD* = 3.03
	English (L2)	N = 133 (61 women, 72 men)	*M*_*age*_ = 22.15, *SD* = 5.21
Familiarity	Polish (L1)	N = 103 (82 women, 21 men)	*M*_*age*_ = 21.74, *SD* = 3.33
	English (L2)	N = 101 (55 women, 46 men)	*M*_*age*_ = 22.94, *SD* = 5.46
Metaphoricity	Polish (L1)	N = 101 (83 women, 18 men)	*M*_*age*_ = 21.27, *SD* = 2.89
	English (L2)	N = 102 (59 women, 43 men)	*M*_*age*_ = 22.15, *SD* = 5.21

For the normative studies on meaningfulness, metaphoricity, and familiarity, analyses of variance (*ANOVAs*) were conducted, whose results are reported below. Significance values for pairwise comparisons were corrected for multiple comparisons using the Bonferroni correction. When Mauchly’s tests showed that the assumption of sphericity was violated, the Greenhouse-Geisser correction was applied. In such cases, the original degrees of freedom are reported with the corrected *p* value.

### Normative studies

With a view to ensuring that all of the critical words were abstract, raters were asked to complete a web-based survey, in which they rated 76 abstract critical nouns together with 76 concrete filler words on a 7-point Likert scale, where 1 represented abstract words and 7 concrete items. The results of paired samples *t*-tests showed that Polish critical words (*M* = 3.12, *SD* = .58) were rated as more abstract compared to concrete filler words (*M* = 5.75, *SD* = .75), *p* < .001. Similarly, English critical words (*M* = 2.96, *SD* = .91) were assessed as more abstract than concrete filler words (*M* = 5.72, *SD* = .65), *p* < .001.

Cloze probability tests were conducted in order to ensure that all of the critical words were not expected due to the preceding context. Raters were provided with a prime word (a verb), and were asked to write a critical word (a noun) which first came to their mind, so that the two-word expression would be meaningful. The final list of stimuli included only those word pairs whose critical words were elicited less than three times in the cloze probability test. Table 4 summarizes the results obtained from the cloze probability tests.

**Table 4:** Cloze probability results for novel metaphoric, conventional metaphoric, literal, and anomalous word dyads in Polish and English.

Language	Novel metaphors	Conventional metaphors	Literal utterances	Anomalous utterances
Polish	0%– 2.86% *(M* = .07%, *SD* = .47)	0%– 2.86% *(M* = .07%, *SD* = .47)	0%– 2.86% *(M* = .26%, *SD* = 1.69)	*M* = 0%, *SD* = 0
English	*M* = 0%, *SD* = 0	*M* = 0%, *SD* = 0	0%– 8.57% *(M* = .64%, *SD* = 2.97)	*M* = 0%, *SD* = 0

To assess the meaningfulness of the word dyads, raters were asked to evaluate their meaningfulness on a scale from 1 (totally meaningless) to 7 (totally meaningful). The analysis conducted on Polish survey results showed a main effect of utterance type, *F*(3, 399) = 1204.32, *p* < .001, ε = .666, η_p_^2^ = .90. Pairwise comparisons further revealed that literal word pairs (*M* = 5.63, *SE* = .06) were rated as more meaningful than conventional metaphors (*M* = 5.44, *SE* = .07), *p* < .001, conventional metaphors were rated as more meaningful than novel metaphors (*M* = 3.70, *SE* = .08), *p* < .001, and novel metaphors were assessed as more meaningful compared to anomalous word pairs (*M* = 1.89, *SE* = .06), *p* < .001. Similarly, the results obtained from English surveys revealed a main effect of utterance type, *F*(3, 387) = 1611.54, *p* < .001, ε = .799, η_p_^2^ = .93. Pairwise comparisons further showed that literal expressions (*M* = 5.99, *SE* = .05) were rated as more meaningful than conventional metaphors (*M* = 5.17, *SE* = .06), *p* < .001, conventional metaphors were rated as more meaningful than novel metaphors (*M* = 4.09, *SE* = .08), *p* < .001, and novel metaphors were assessed as more meaningful compared to anomalous utterances (*M* = 2.33, *SE* = .06), *p* < .001.

With a view to examining the familiarity of the stimuli, raters were asked to decide how often they encountered the presented word pairs on a scale from 1 (very rarely) to 7 (very frequently). Based on the results obtained from the surveys conducted on Polish stimuli, the analysis revealed a main effect of utterance type, *F*(2, 200) = 684.63, *p* < .001, ε = .920, η_p_^2^ = .87. Pairwise comparisons confirmed that novel metaphors (*M* = 2.39, *SE* = .07) were less familiar than both literal expressions (*M* = 4.10, *SE* = .09), *p* < .001, and conventional metaphors (*M* = 4.28, *SE* = .08), *p* < .001. Furthermore, literal expressions were less familiar than conventional metaphors, *p* = .002. With respect to Likert scales on English word dyads, a main effect of utterance type was found, *F*(2, 296) = 470.97, *p* < .001, ε = .801, η_p_^2^ = .83. Pairwise comparisons showed that English novel metaphors (*M* = 2.15, *SE* = .07) were rated as less familiar than conventional metaphors (*M* = 2.97, *SE* = .08), *p* < .001, as well as than literal expressions (*M* = 3.85, *SE* = .09), *p* < .001. Furthermore, conventional metaphors were less familiar than literal word dyads, *p* < .001.

In order to evaluate the metaphoricity of the stimuli, raters were asked to decide how metaphorical given word dyads were on a scale from 1 (very literal) to 7 (very metaphorical). The analysis carried out on the results from Polish surveys showed a main effect of utterance type, *F*(2, 196) = 605.55, *p* < .001, ε = .733, η_p_^2^ = .86. Pairwise comparisons further showed that novel metaphors (*M* = 5.47, *SE* = .06) were judged as more metaphorical than conventional metaphors (*M* = 4.05, *SE* = .08), *p* < .001, and conventional metaphors were rated as more metaphorical than literal word dyads (*M* = 2.65, *SE* = .09), *p* < .001. The results obtained from English surveys also revealed a main effect of utterance type, *F*(2, 198) = 588.82, *p* < .001, ε = .738, η_p_^2^ = .86. Pairwise comparisons confirmed that novel metaphors (*M* = 5.00, *SE* = .06) were rated as more metaphorical than conventional metaphors (*M* = 3.98, *SE* = .06), *p* < .001, and conventional metaphors were rated as more metaphorical than literal utterances (*M* = 2.74, *SE* = .07), *p* < .001.

The aforementioned normative studies resulted in the final list of 304 Polish and 304 English word pairs that were used in the ERP experiment. The results of the normative studies for those word dyads that were included in the final list are reported in Table 5. For the EEG experiment, the materials were split into 8 blocks (4 in Polish and 4 in English) in order to avoid the repetition of critical words within one block. Each block consisted of 19 novel metaphors, 19 conventional metaphors, 19 literal, and 19 anomalous expressions. Additionally, 30 filler word dyads were added to each block. The filler word pairs differed from experimental stimuli in their syntactic structure, and instead of containing verb-noun phrases, they included adjective-noun or noun-noun word dyads. Furthermore, in order to balance out the number of positive and negative responses to the utterances, all of the filler word dyads were meaningless. Altogether, 106 randomized expressions were used in each block. Each participant completed all 8 blocks, which were presented in a randomized order.

**Table 5:** Results of the normative studies on stimuli included in the experiment.

Utterance type	Meaningfulness		Familiarity		Metaphoricity	
	Polish	English	Polish	English	Polish	English
Novel metaphors	*M* = 3.70, *SE* = .08	*M* = 4.09, *SE* = .08	*M* = 2.39, *SE* = .07	*M* = 2.15, *SE* = .07	*M* = 5.47, *SE* = .06	*M* = 5.00, *SE* = .06
Conventional metaphors	*M* = 5.44, *SE* = .07	*M* = 5.17, *SE* = .06	*M* = 4.28, *SE* = .08	*M* = 2.97, *SE* = .08	*M* = 4.05, *SE* = .08	*M* = 3.98, *SE* = .06
Literal utterances	*M* = 5.63, *SE* = .06	*M* = 5.99, *SE* = .05	*M* = 4.10, *SE* = .09	*M* = 3.85, *SE* = .09	*M* = 2.65, *SE* = .09	*M* = 2.74, *SE* = .07
Anomalous utterances	*M* = 1.89, *SE* = .06	*M* = 2.33, *SE* = .06	-	-	-	-

### Procedure

The procedures applied in the experiment were in accordance with the ethical guidelines for research with human participants, and were approved by the Adam Mickiewicz University Human Research Ethics Committee. Participants were informed about the procedures of the experiment, and were asked to sign the informed consent form before the experiment began.

**Fig 1:**
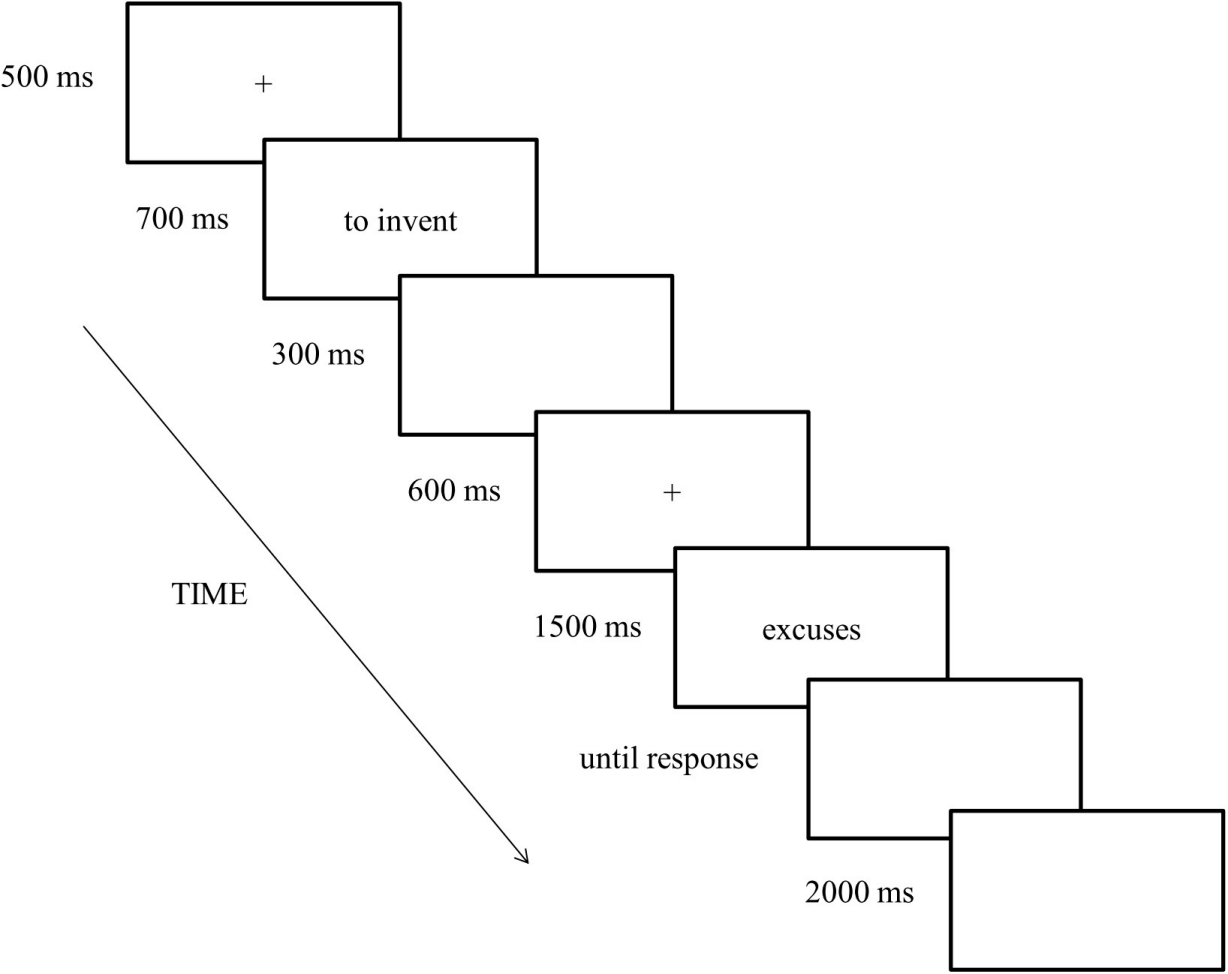
Time sequence of stimulus presentation.

The word dyads were randomly presented, one word at a time, on a computer screen using black letters, and were centered on a gray background. The expressions were presented in the following time sequence: the first fixation cross (500 ms) was followed by a prime word (700 ms), a blank screen (300 ms), the second fixation cross (600 ms), and finally a critical word (1500 ms). After the critical word, a blank screen was displayed until participants made their decisions. Trials were separated by an intertrial interval (2000 ms), which was presented as a blank screen. The time sequence of stimulus presentation is provided in Fig 1.

Participants were instructed to decide whether the word dyad displayed was meaningful or meaningless, and to press a corresponding key, whose designation was counterbalanced between participants. Prior to the experimental blocks, participants completed a practice block with 20 two-word expressions not included in the experimental trials, in order to practice the task. Participants who began the experiment with English blocks were instructed in English, and those who started the experiment with Polish blocks were provided with Polish instructions.

### Electrophysiological recording

EEG signals were recorded from 64 active Ag/AgCl electrodes (Brain Products): FP1, FP2, F7, F3, Fz, F4, F8, FC5, FC1, FC2, FC6, T7, C3, C2, C4, T8, TP9, CP5, CP1, CP2, CP6, TP10, P7, P3, Pz, P4, P8, PO9, O1, Oz, O2, PO10, AF7, AF3, AF4, AF8, F5, F1, F2, F6, FPz, FT7, FC3, FC4, FT8, FCz, C5, C1, Cz, C6, TP7, CP3, CPz, CP4, TP8, P5, P1, P2, P6, PO7, PO3, POz, PO4, PO8 at the standard extended 10/20 positions with the ground placed at AFz. To monitor vertical eye movements, bipolar electrodes were placed above and below the right eye (vEOG). For horizontal eye movements, bipolar electrodes were placed horizontally from positions next to the outer rims of the eyes (hEOG). EEG signal was amplified by the QuickAmp amplifier (BrainProducts GmbH) with average reference, filtered with low-pass (cutoff frequency = 0.016 Hz), and stored at 1000 Hz per channel. All data were stored on a computer for offline analyses. Impedances were kept below 5 kΩ for each electrode. ERPs were time-locked to the onset of the second word of the expression.

The electrodes selected for the statistical analyses included: FC1, FC3, FCz, FC2, FC4, C1, C3, Cz, C2, C4, CP1, CP3, CPz, CP2, CP4, P1, P3, Pz, P2, P4. Offline data analyses were conducted using BrainVision Analyzer 2.0 software. Data were segmented from 100 ms before stimulus onset to 900 ms afterward, and filtered with Butterworth Zero Phase Filter (low cutoff: 0.5 Hz, high cutoff: 30 Hz); 24 dB/oct slope was used. Furthermore, data were referred to baseline -100 to 0 ms before stimulus onset, and edited for artifacts (rejecting trials with zero lines, rejecting trials with voltage differences higher than 150 μV or voltage steps higher than 50 μV). Ocular artifacts were rejected by the Gratton & Coles method.

For the peak detection analysis, averaged amplitudes for all electrodes over which the N400 amplitude difference between L1 and L2 was most pronounced (i.e., C3, C1, CP3, CP1, P3, P1 electrodes) were computed separately for Polish and English. The analysis was performed in a semi-automatic mode, using a detection method based on global maxima in the predefined interval of 300–500 ms post stimulus onset. Once the N400 peaks for L1 and L2 were detected, a paired samples *t*-test was performed on the N400 peak latencies to Polish and English utterances.

### Overview of statistical analyses

Both accuracy ratings and reaction times were analyzed using 2 language (Polish vs. English) × 4 utterance type (novel vs. conventional vs. literal vs. anomalous) repeated measures *ANOVAs*. With respect to event-related potentials, mean amplitudes from 20 electrodes for each condition in each block were selected for the analysis. Along the anterior-posterior axis, the following electrodes were chosen: FC3, FC1, FCz, FC2, FC4 (fronto-central), C3, C1, Cz, C2, C4 (central), CP3, CP1, CPz, CP2, CP4 (centro-parietal), P3, P1, Pz, P2, P4 (parietal). Along the left-right axis, the following electrodes were selected: FC3, C3, CP3, P3 (left), FC1, C1, CP1, P1 (left medial), FCz, Cz, CPz, Pz (midline), FC4, C4, CP4, P4 (right), FC2, C2, CP2, P2 (right medial). Statistical analyses were performed between 100–900 ms after stimulus onset. To avoid possible bias caused by brain activity associated with correct responses rather than a given stimulus category [56], all responses were used in the analysis. Additionally, the observed effects did not differ from those found in the analysis based on correctly categorized trials. Based on visual inspection, three clear peaks were observed, and the following respective time windows were selected for the ERP analysis: the 150–250 ms time window (P200), the 300–500 ms time window (N400), and the 500–800 ms time window (LPC). These time windows are also in line with previous literature on these components. Additionally, visual inspection indicated that the N400 amplitudes for individual utterance types converged between 300–400 ms, and started to diverge at around 400 ms. For this reason, the N400 time window was divided into the early (300–400 ms) and late (400–500 ms) N400 time frame. Such an approach has previously been applied in ERP research on metaphoric meaning comprehension [29].

Mean amplitudes were analyzed using 2 language (Polish/native dominant vs. English/non-native non-dominant) × 4 utterance type (novel vs. conventional vs. literal vs. anomalous) × 4 anterior-posterior electrode position (fronto-central vs. central vs. centro-parietal vs. parietal) × 5 laterality (left vs. left medial vs. midline vs. right medial vs. right) × 4 block order (first vs. second vs. third vs. fourth) repeated measures *ANOVAs*. In all analyses, significance values for pairwise comparisons were corrected for multiple comparisons using the Bonferroni correction. When Mauchly’s tests showed that the assumption of sphericity was violated, the Greenhouse-Geisser correction was applied. In such cases, the original degrees of freedom are reported with the corrected *p* value.

## Results

### Behavioral results

#### Accuracy rates

Accuracy ratings are reported as percentage of correct responses observed in the semantic decision task. The statistical analysis revealed an interaction between language and utterance type, *F*(3, 66) = 21.38, *p* < .001, ε = .569, η_p_^2^ = .493. Follow up analyses were carried out for each language separately. A repeated measures *ANOVA* with utterance types as factor performed on accuracy rates for Polish utterances showed a main effect of utterance type, *F*(3, 66) = 72.61, *p* < .001, ε = .426, η_p_^2^ = .767. Pairwise comparisons confirmed that novel metaphors (*M* = 48.85, *SE* = 3.89) were rated less accurately than conventional metaphors (*M* = 85.58, *SE* = 2.16), *p* < .001, as well as literal meanings (*M* = 90.10, *SE* = 1.29), *p* < .001, and anomalous utterances (*M* = 92.85, *SE* = 1.89), *p* < .001. There was no statistically significant difference between conventional metaphoric and literal, between conventional metaphoric and anomalous, as well as between literal and anomalous word pairs, *p*s > .05.

Similarly, a repeated measures *ANOVA* with utterance types as factor performed on accuracy rates for English utterances showed a main effect of utterance type, *F*(3, 66) = 49.13, *p* < .001, ε = .389, η_p_^2^ = .691. Pairwise comparisons revealed that novel metaphors (*M* = 44.79, *SE* = 3.87) differed from conventional metaphors (*M* = 62.87, *SE* = 2.52), *p* < .001, from literal utterances (*M* = 84.90, *SE* = 1.39), *p* < .001, as well as from anomalous utterances (*M* = 86.90, *SE* = 2.93), *p* < .001. Additionally, conventional metaphors differed from literal, *p* < .001, and from anomalous word pairs, *p* < .001. There was no statistically significant difference between literal and anomalous utterances, *p* > .05.

With regard to between language differences, post-hoc tests revealed that conventional metaphors were rated more accurately in Polish than in English, *p* < .001, and the same pattern of results was observed for Polish and English literal expressions, *p* = .002, and Polish and English anomalous utterances, *p* = .001. There was no statistically significant difference between Polish and English novel metaphors, *p* > .05. Correction for multiple comparisons was applied here, and the critical *p* level for significance was set to .012.

**Fig 2:**
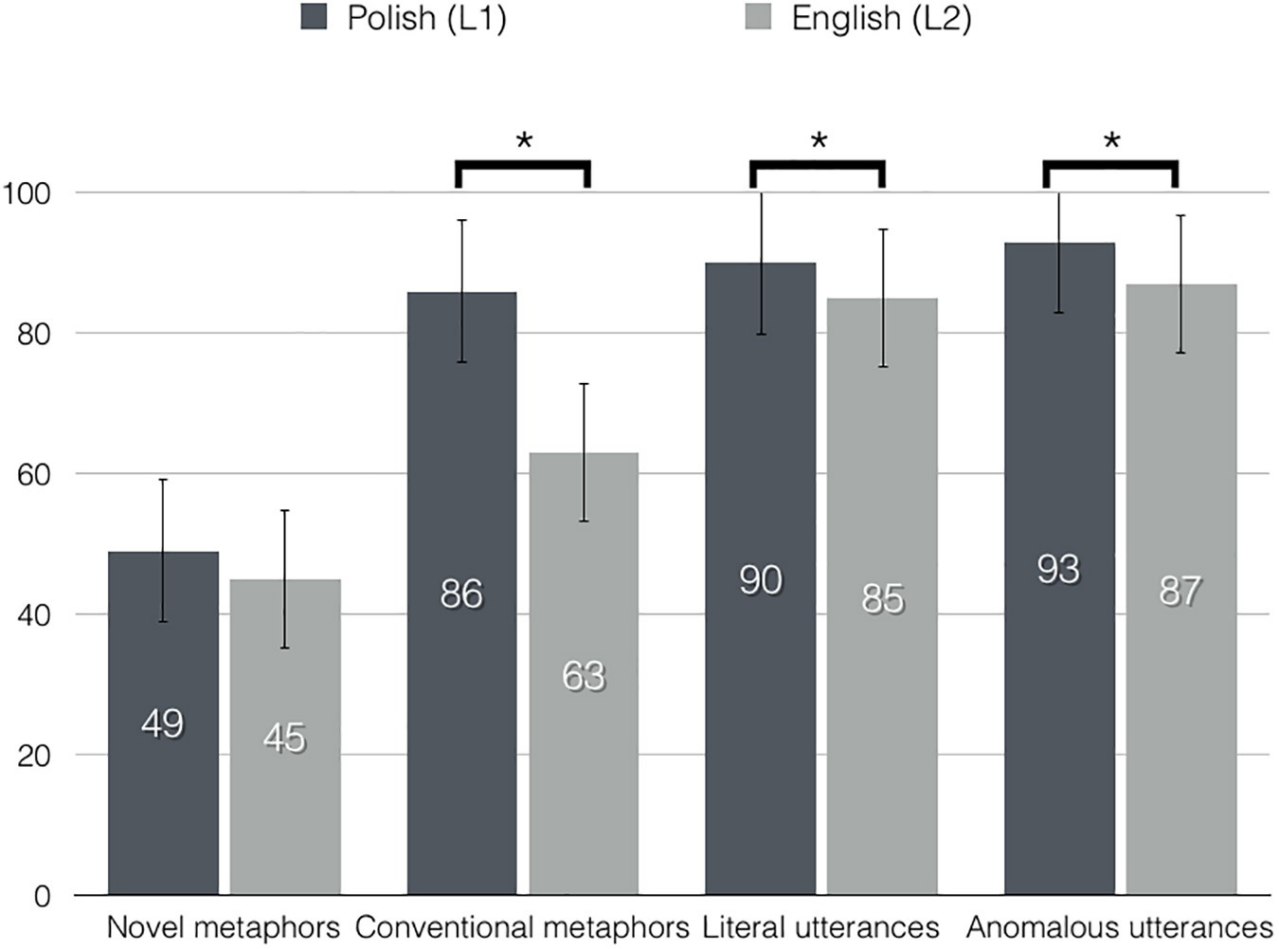
Accuracy rates (%) to novel metaphoric, conventional metaphoric, literal, and anomalous word dyads in Polish (dark grey) and English (light grey).

In addition to the interaction, a main effect of language was found, *F*(1, 22) = 60.89, *p* < .001, η_p_^2^ = .735. The accuracy rate was higher for Polish expressions (*M* = 79.35, *SE* = 1.38) than for English utterances (*M* = 69.87, *SE* = 1.38). Furthermore, a main effect of utterance type was found, *F*(3, 66) = 64.65, *p* < .001, ε = .388, η_p_^2^ = .746. Pairwise comparisons confirmed that novel metaphors (*M* = 46.82, *SE* = 3.62) differed from conventional metaphors (*M* = 74.23, *SE* = 2.05), *p* < .001, from literal utterances (*M* = 87.50, *SE* = 1.18), *p* < .001, as well as from anomalous utterances (*M* = 89.87, *SE* = 2.34), *p* < .001. Furthermore, conventional metaphors differed from literal, *p* < .001, and from anomalous utterances, *p* = .004. There was no statistically significant difference between literal and anomalous word dyads, *p* > .05. Mean accuracy rates per each utterance type in each language are provided in Fig 2.

**Fig 3:**
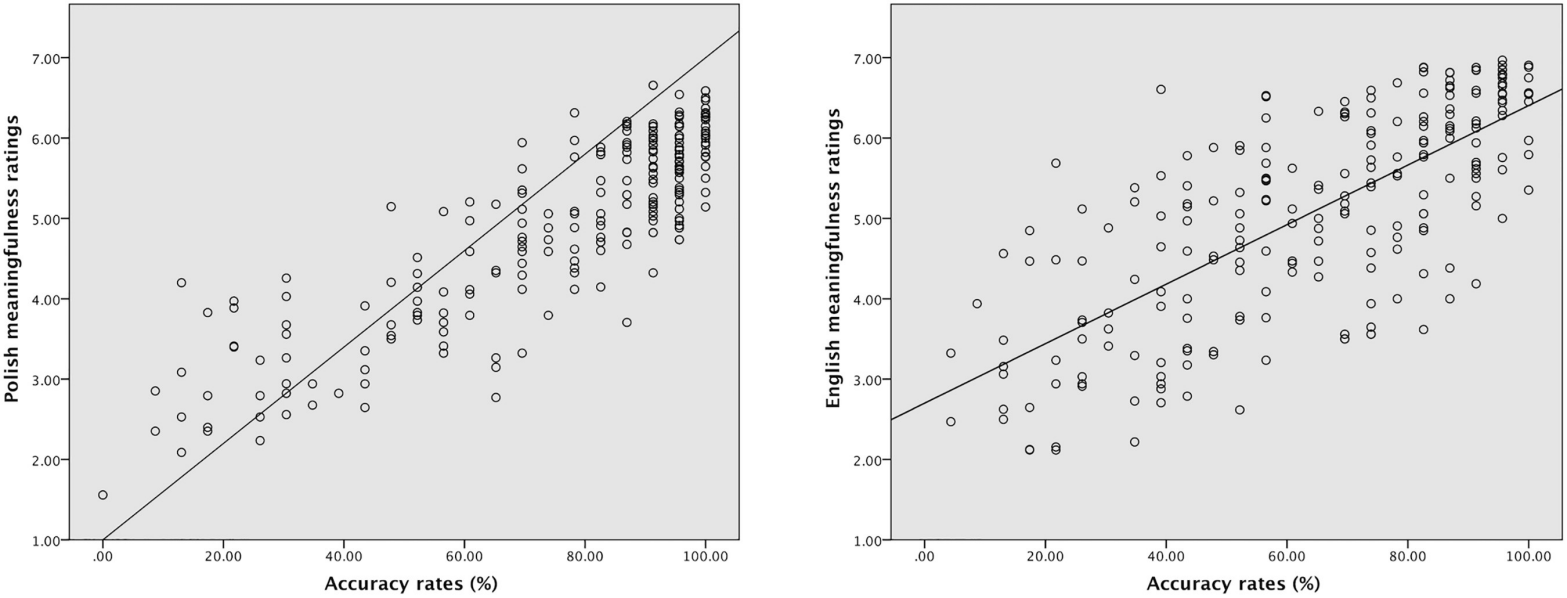
Scatterplots showing the correlations between meaningfulness ratings (y-axis) and accuracy rates (x-axis) for word dyads in Polish (left-hand side) and English (right-hand side).

Since accuracy rates for Polish stimuli were higher than for English materials, a correlation analysis was conducted in order to check whether the word dyads were evaluated similarly by the normative study participants (native speakers of Polish and English) and the EEG experiment participants (native speakers of Polish and L2 learners of English). The results showed a strong positive correlation between accuracy rates for novel metaphoric, conventional metaphoric, and literal word pairs in the EEG experiment and in the normative meaningfulness ratings for both Polish (*r*_*s*_ = .813, *p* < .001) and English word dyads (*r*_*s*_ = .731, *p* < .001). In English, the correlations for literal utterances (*r*_*s*_ = .594, *p* < .001), conventional metaphors (*r*_*s*_ = .573, *p* < .001), and novel metaphors (*r*_*s*_ = .502, *p* < .001) were comparably strong. In Polish, on the other hand, the correlation was most robust for novel metaphors (*r*_*s*_ = .749, *p* < .001), while for conventional metaphors (*r*_*s*_ = .549, *p* < .001) and literal word pairs (*r*_*s*_ = .537, *p* < .001) the correlations were comparably strong. Scatterplots showing the correlations between meaningfulness ratings and accuracy rates for Polish and English stimuli are presented in Fig 3.

#### Reaction times

Reaction times (RTs) were measured time-locked to critical word onset. Only correct responses were included in the statistical analysis. The results showed an interaction between language and utterance type, *F*(3, 66) = 6.25, *p* = .001, η_p_^2^ = .221. Follow up analyses were carried out for each language separately. A repeated measures *ANOVA* with utterance types as factor performed on reaction times for Polish utterances further revealed a main effect of utterance type, *F*(3, 66) = 14.68, *p* < .001, ε = .698, η_p_^2^ = .400. Pairwise comparisons confirmed that novel metaphors (*M* = 1121.25 ms, *SE* = 77.17) elicited significantly longer RTs than conventional metaphors (*M* = 986.84 ms, *SE* = 79.19), *p* < .001, literal utterances (*M* = 988.44 ms, *SE* = 80.87), *p* < .001, and anomalous utterances (*M* = 987.06 ms, *SE* = 73.05), *p* = .003. There was no statistically significant difference between conventional metaphoric and literal, between conventional metaphoric and anomalous, as well as between literal and anomalous word pairs, *p*s > .05.

Similarly, a repeated measures *ANOVA* with utterance types as factor performed on reaction times for English utterances showed a main effect of utterance type, *F*(3, 66) = 8.39, *p* = .001, ε = .623, η_p_^2^ = .276. However, pairwise comparisons in English revealed a somewhat different pattern of results than in Polish. Namely, English novel metaphors elicited longest RTs (*M* = 1189.73 ms, *SE* = 87.09), which were however similar to those evoked by conventional metaphors (*M* = 1155.19 ms, *SE* = 92.36) and anomalous word pairs (*M* = 1113.68 ms, *SE* = 86.48), *p*s > .05. Shortest reaction times were elicited by literal word pairs (*M* = 1090.07 ms, *SE* = 85.87), which differed significantly from both novel, *p* < .001, and conventional metaphors, *p* = .003. There was no statistically significant difference between anomalous and literal, and between anomalous and conventional metaphoric word pairs, *p*s > .05.

With regard to between language differences, paired samples *t*-tests showed that English conventional metaphors differed from Polish conventional metaphors, *p* = .001. There was no statistically significant difference between Polish and English novel metaphoric, literal, as well as anomalous word pairs, *p* > .05. Correction for multiple comparisons was applied here, and the critical *p* level for significance was set to .012.

**Fig 4:**
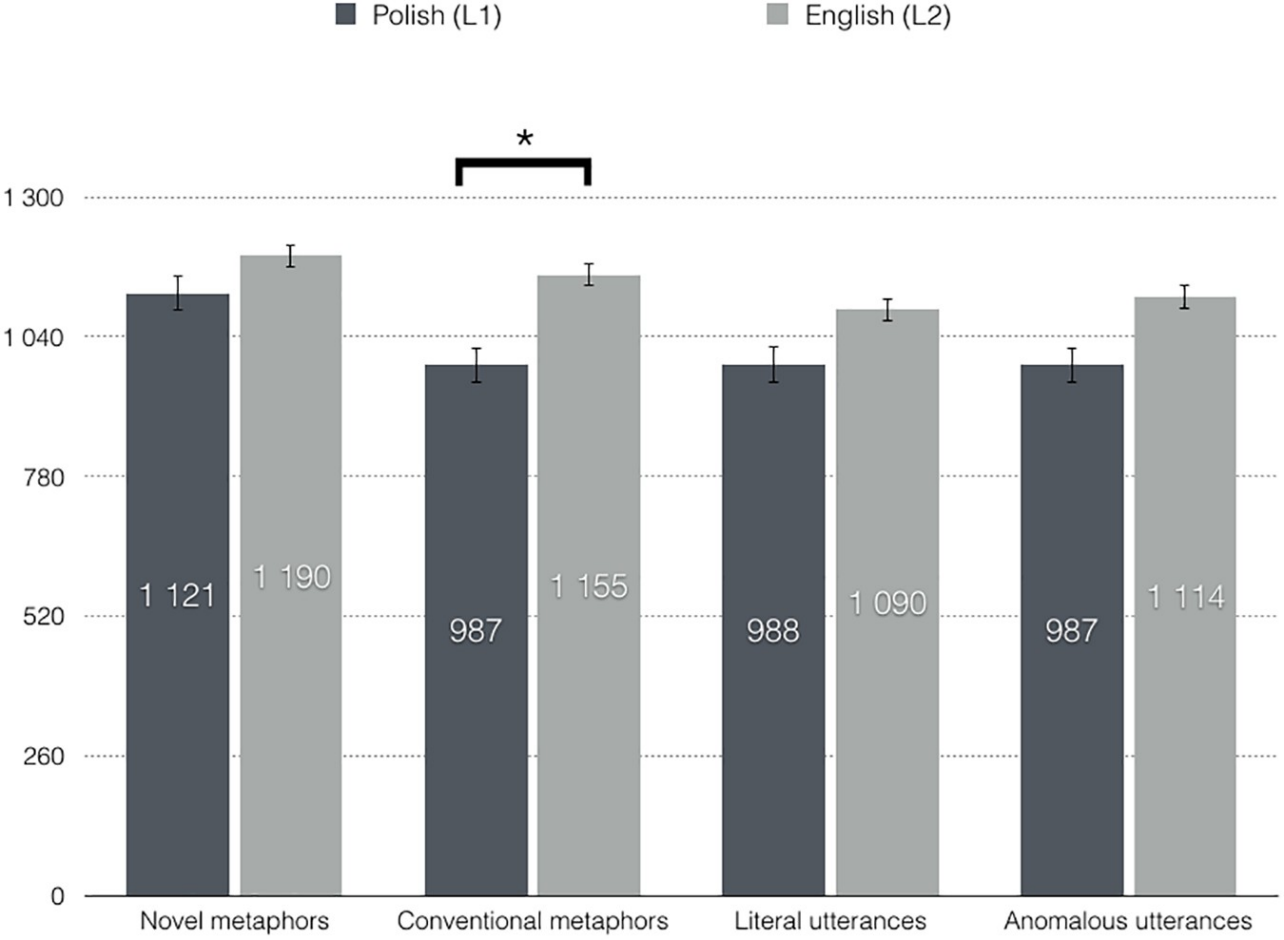
Reaction times (ms) to novel metaphoric, conventional metaphoric, literal, and anomalous word dyads in Polish (dark grey) and English (light grey).

In addition to the interaction, a main effect of language was observed, *F*(1, 22) = 6.95, *p* = .015, η_p_^2^ = .240, with longer reaction times elicited by English (*M* = 1137.17 ms, *SE* = 86.94) than Polish expressions (*M* = 1020.90 ms, *SE* = 76.14). Moreover, a main effect of utterance type was found, *F*(3, 66) = 12.19, *p* < .001, ε = .656, η_p_^2^ = .389. Pairwise comparisons confirmed that novel metaphors (*M* = 1155.49 ms, *SE* = 79.00) elicited longer RTs compared to literal utterances (*M* = 1039.26 ms, *SE* = 80.54), *p* < .001, conventional metaphors (*M* = 1071.02 ms, *SE* = 83.20), *p* = .001, and anomalous utterances (*M* = 1050.37 ms, *SE* = 75.58), *p* = .007. There was no statistically significant difference between anomalous and literal, between anomalous and conventional metaphoric, as well as between literal and conventional metaphoric word dyads, *p*s > .05. Mean reaction times per each utterance type in each language are provided in Fig 4.

### Event-related potentials

#### 150–250 ms, P200

**Fig 5:**
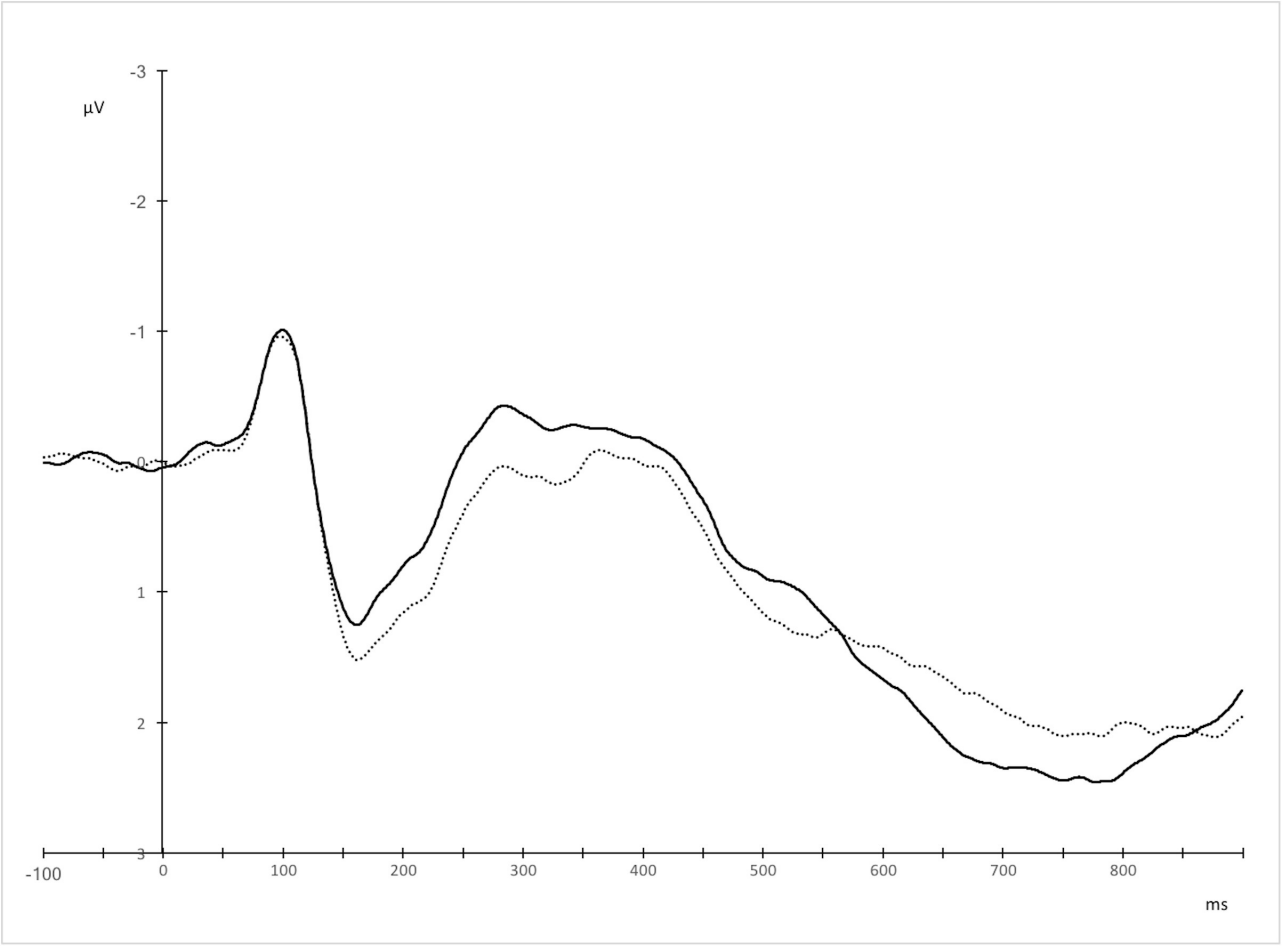
Grand averages for Polish (solid line) and English (dotted line) utterances over left central and centro-parietal electrodes, where the effect was maximal.

**Fig 6:**
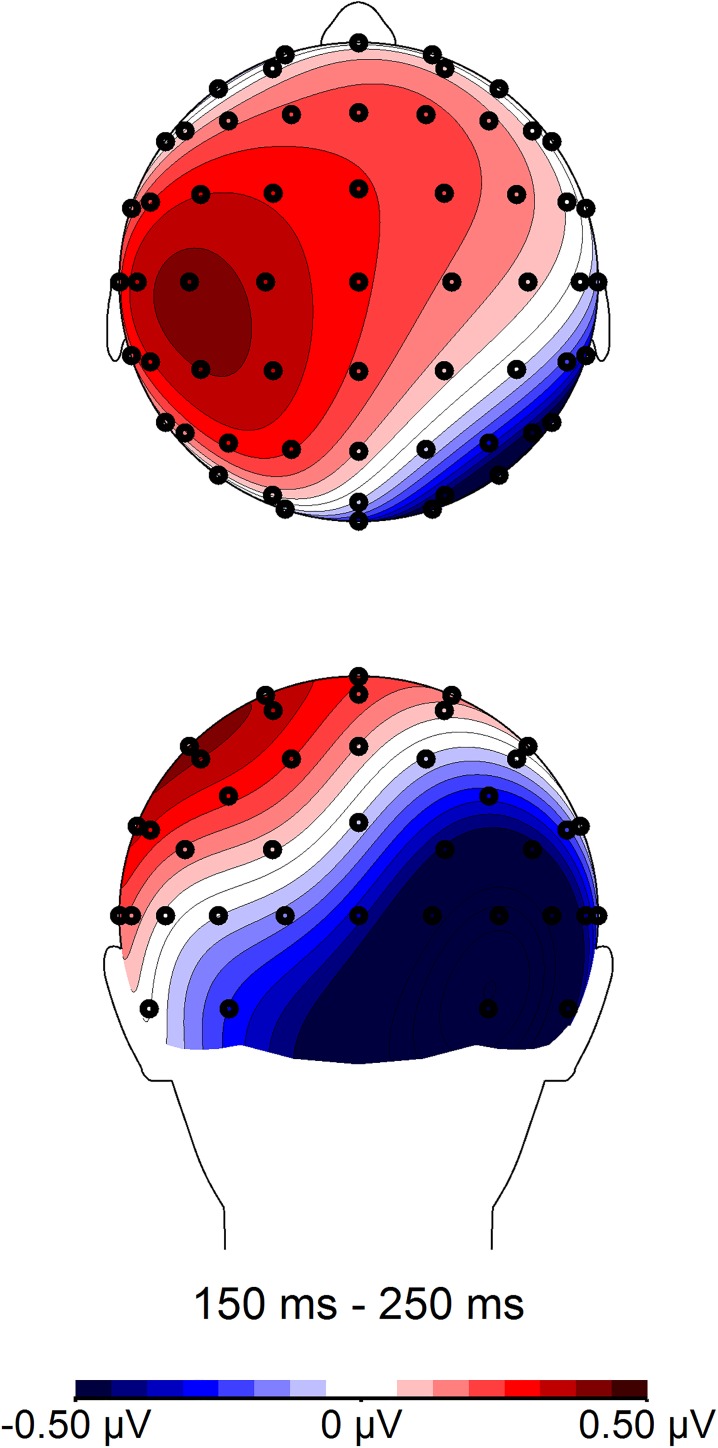
Topographic distribution of Polish and English word dyads in the 150–250 ms time window. Voltage maps were obtained for the averaged value of difference waves (English word pairs minus Polish word pairs).

Within the 150–250 ms time window, an interaction was found between anterior-posterior electrode position, laterality, and language, *F*(12, 264) = 2.33, *p* = .048, ε = .408, η_p_^2^ = .096. Furthermore, within the same time window, an interaction was observed between laterality and language, *F*(4, 88) = 12.38, *p* < .001, ε = .558, η_p_^2^ = .360. Block order did not interact with either utterance type or language within the P200 time window, and such interactions did not emerge within the N400 and LPC time frames. To deconstruct the interactions, we computed averaged amplitudes for all left (FC3, FC1, C3, C1, CP3, CP1, P3, P1) and all right electrodes (FC2, FC4, C2, C4, CP2, CP4, P2, P4) separately for fronto-central, central, centro-parietal, and parietal electrode positions. For each of these levels of the anterior-posterior axis, a 2 language (Polish vs. English) × 2 laterality (left vs. right) repeated measures *ANOVA* was performed. An interaction between language and laterality was observed over central, centro-parietal, and parietal electrodes, but not over fronto-central electrodes (Table 6). To test whether between-language differences would be more pronounced over left or right sites, we performed paired samples *t*-tests on amplitudes for English and Polish utterances averaged separately for left central, left centro-parietal, left parietal, right central, right centro-parietal, and right parietal electrodes (Table 7). The results showed maximal between-language differences over left central and left centro-parietal electrodes, and less pronounced over left parietal electrodes, with reduced P200 amplitudes for Polish compared to English word pairs (Fig 5, Fig 6). No between-language differences were observed over right sites, *p*s > .05.

**Table 6:** An interaction between language and laterality over fronto-central, central, centro-parietal, and parietal electrode positions within the 150–250 ms time window.

Electrode position	An interaction between language and laterality
Fronto-central	*F*(4, 88) = .97, *p* = .402, η_p_^2^ = .042
Central	*F*(4, 88) = 8.00, *p* < .001, η_p_^2^ = .267
Centro-parietal	*F*(4, 88) = 10.46, *p* < .001, η_p_^2^ = .322
Parietal	*F*(4, 88) = 9.08, *p* < .001, η_p_^2^ = .292

**Table 7:** Between-language differences within the 150–250 ms time window, with mean amplitudes for Polish and English utterances over left and right central, centro-parietal, and parietal electrode positions.

Electrode position		Mean amplitude for Polish utterances	Mean amplitude for English utterances	Between-language difference
Central	Left (C3, C1)	*M* = 1.278, *SE* = .213	*M* = 1.661, *SE* = .228	*t*(22) = 4.33, *p* < .001, *r* = .68
	Right (C4, C2)	*M* = 1.345, *SE* = .168	*M* = 1.470, *SE* = .195	*t*(22) = 1.39, *p* = .178, *r* = .28
Centro-parietal	Left (CP3, CP1)	*M* = .228, *SE* = .183	*M* = .580, *SE* = .229	*t*(22) = 4.31, *p* < .001, *r* = .68
	Right (CP4, CP2)	*M* = .448, *SE* = .187	*M* = .478, *SE* = .187	*t*(22) = .31, *p* = .76, *r* = .06
Parietal	Left (P3, P1)	*M* = -.697, *SE* = .185	*M* = -.420, *SE* = .222	*t*(22) = 2.47, *p* = .022, *r* = .47
	Right (P4, P2)	*M* = .048, *SE* = .287	*M* = -.135, *SE* = .336	*t*(22) = -1.194, *p* = .245, *r* = .24

#### 300–400 ms, the early N400

The analysis of the early N400 time window revealed an interaction between anterior-posterior electrode position and language, *F*(3, 66) = 5.12, *p* = .027, ε = .394, η_p_^2^ = .189, and an interaction between laterality and language, *F*(4, 88) = 14.60, *p* < .001, ε = .522, η_p_^2^ = .399. To deconstruct the interactions, we computed averaged amplitudes for all left and all right electrodes separately for fronto-central, central, centro-parietal, and parietal electrode positions, similarly to the analyses performed within the P200 time frame. For each of these levels of the anterior-posterior axis, a 2 language (Polish vs. English) × 2 laterality (left vs. right) repeated measures *ANOVA* was performed. These analyses revealed a main effect of language over central, centro-parietal, and parietal electrodes, where English utterances evoked attenuated N400 responses compared to Polish word dyads (Table 8).

**Table 8:** Main effect of language within the 300–400 ms time window, with mean amplitudes for Polish and English utterances over fronto-central, central, centro-parietal, and parietal electrode positions.

Electrode position	Mean amplitude for Polish utterances	Mean amplitude for English utterances	Main effect of language
Fronto-central	*M* = -.658, *SE* = .336	*M* = -.555, *SE* = .267	*F*(1, 22) = .34, *p* = .568, η_p_^2^ = .015
Central	*M* = -.768, *SE* = .226	*M* = -.390, *SE* = .196	*F*(1, 22) = 6.49, *p* = .018, η_p_^2^ = .228
Centro-parietal	*M* = -.199, *SE* = .241	*M* = .490, *SE* = .219	*F*(1, 22) = 16.46, *p* = .001, η_p_^2^ = .428
Parietal	*M* = .903, *SE* = .337	*M* = 1.633, *SE* = .328	*F*(1, 22) = 20.25, *p* < .001, η_p_^2^ = .479

**Fig 7:**
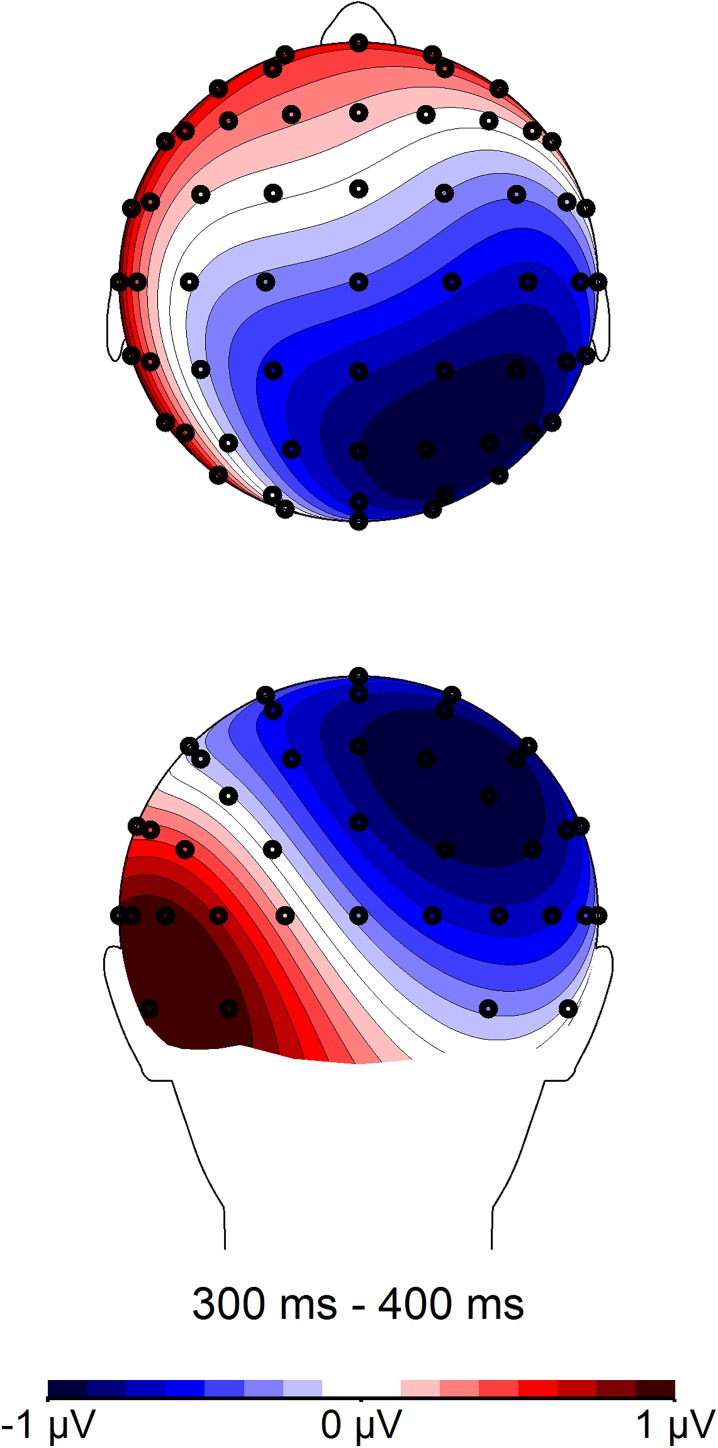
Topographic distribution of Polish and English word dyads in the 300–400 ms time window. Voltage maps were obtained for the averaged value of difference waves (Polish word pairs minus English word pairs).

**Fig 8:**
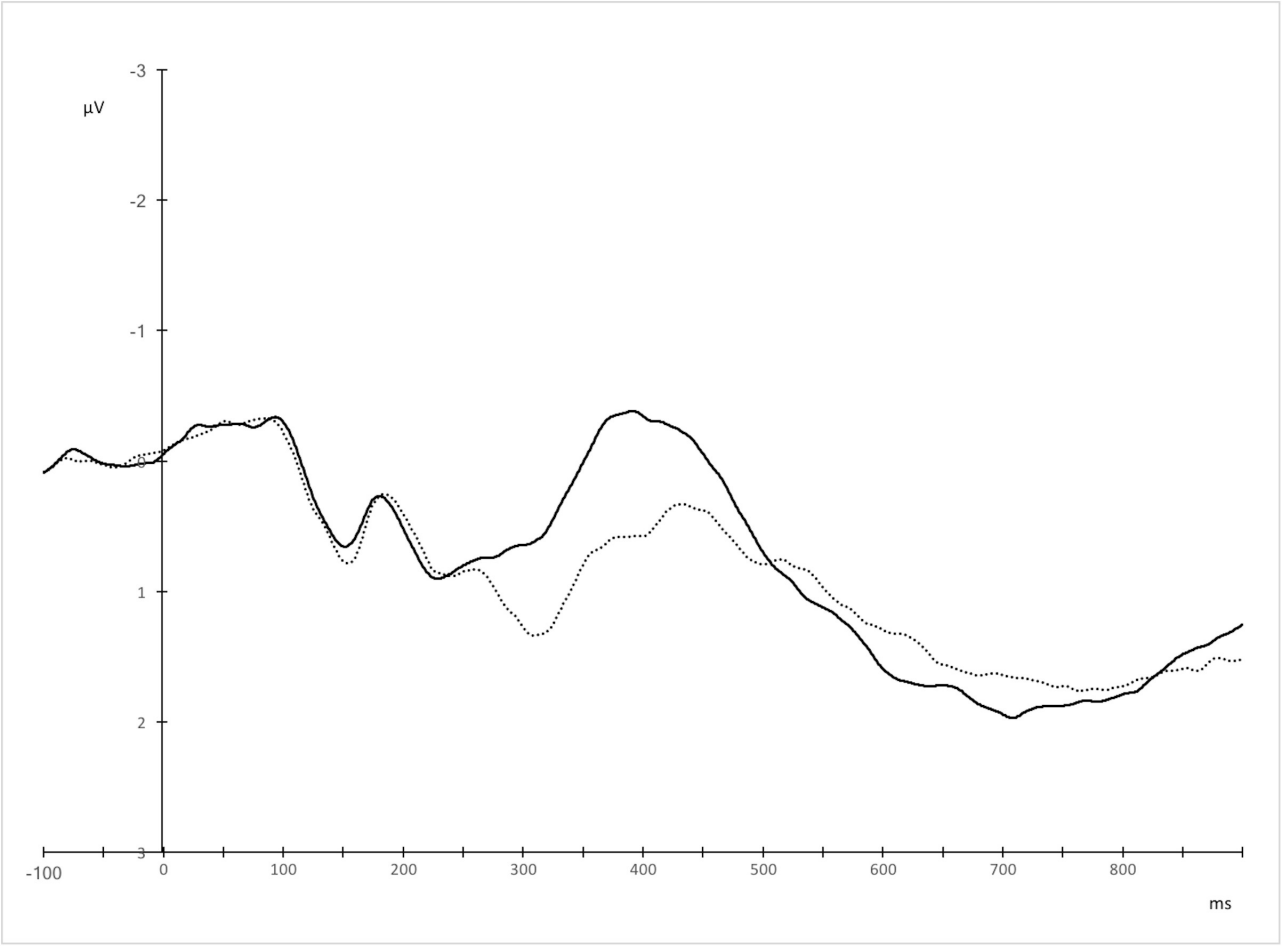
Grand averages for Polish (solid line) and English (dotted line) utterances over right central, centro-parietal, and parietal electrodes, where the effect was maximal.

We further averaged amplitudes across all left and all right electrodes for all central, centro-parietal, and parietal electrode positions. A 2 language (Polish vs. English) × 2 laterality (left vs. right) repeated measures *ANOVA* was performed, and showed an interaction between language and laterality, *F*(1, 22) = 24.11, *p* < .001, η_p_^2^ = .523. To decompose this interaction, paired samples *t*-tests were performed on averaged amplitudes separately for all left central, centro-parietal, and parietal electrodes, as well as all right central, centro-parietal, and parietal electrodes (Table 9, Fig 7, Fig 8). Amplitudes for English utterances were attenuated relative to Polish word pairs. The effect was most pronounced over right electrodes, where the mean difference between amplitudes for English vs. Polish utterances was .828 μV (*r* = .79). Over left sites, the mean difference was much smaller (.305 μV, *r* = .44).

**Table 9:** Between-language differences within the 300–400 ms time window, with mean amplitudes for Polish and English utterances over left and right electrode positions.

Electrode position	Mean amplitude for Polish utterances	Mean amplitude for English utterances	Between-language difference
Left (C3, C1, CP3, CP1, P3, P1)	*M* = .219, *SE* = .199	*M* = .524, *SE* = .204	*t*(22) = -2.31, *p* = .031, *r* = .44
Right (C4, C2, CP4, CP2, P4, P2)	*M* = .062, *SE* = .267	*M* = .900, *SE* = .223	*t*(22) = -6.13, *p* < .001, *r* = .79

To test whether the N400 peak latency to the non-native and non-dominant language (English) was delayed relative to the native and dominant language (Polish), a peak detection analysis was performed. Since differences in the N400 amplitudes between L1 and L2 were most pronounced over right central, centro-parietal, and parietal electrodes (Fig 8), we estimated peak latencies for the averaged waveforms over these electrodes separately for Polish and English. The statistical comparison of these peak latencies between the two languages confirmed a delayed N400 response to L2 (*M* = 424.57, *SD* = 38.93) compared to L1 word dyads (*M* = 406.26, *SD* = 34.02), *t*(22) = -2.15, *p* = .042, *r* = .42.

#### 400–500 ms, the late N400

**Fig 9:**
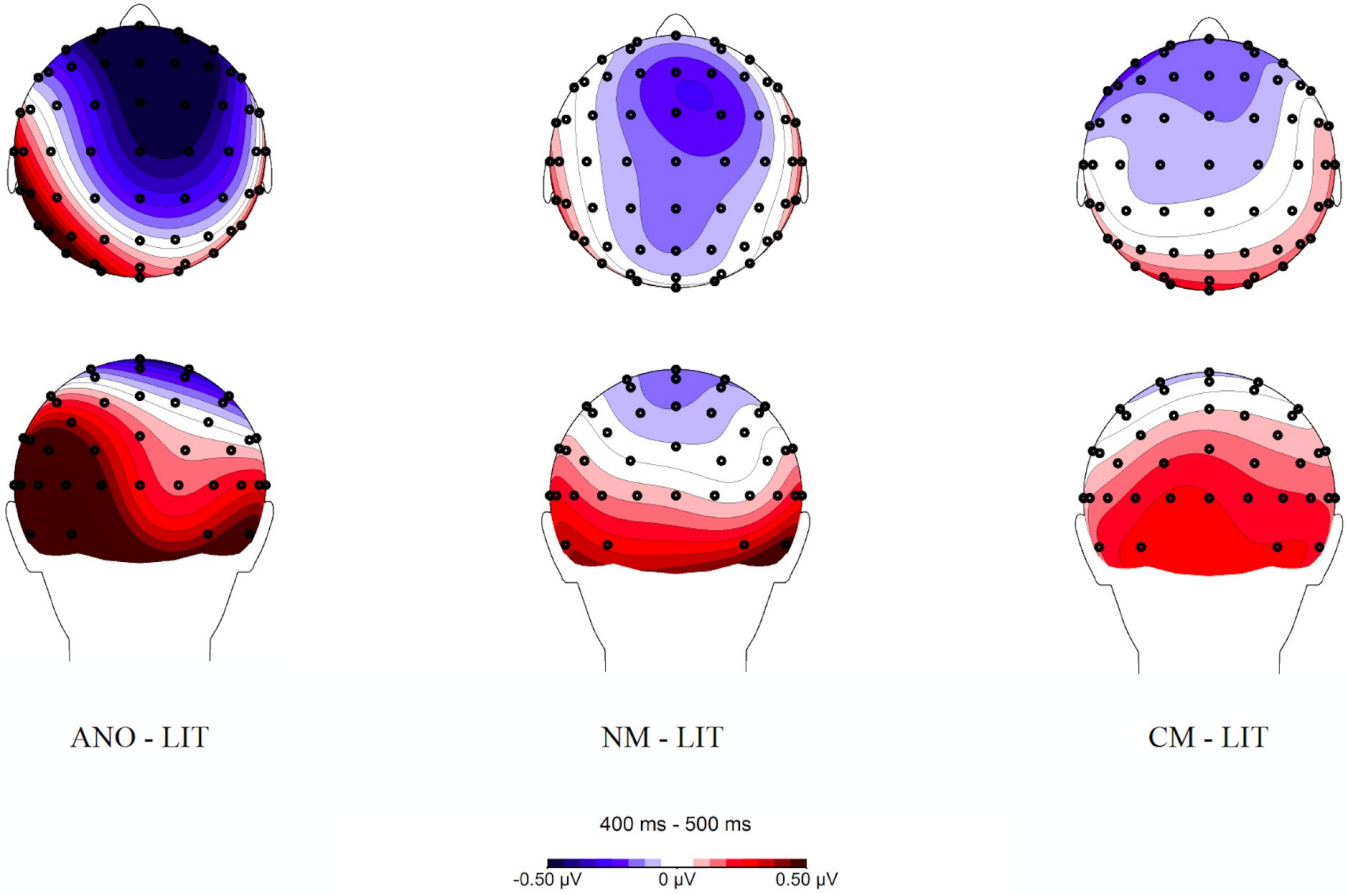
Topographic distribution of novel metaphoric (NM), conventional metaphoric (CM), literal (LIT), and anomalous (ANO) word dyads in the 400–500 ms time window. Voltage maps were obtained for the averaged value of difference waves (anomalous minus literal word pairs, novel metaphoric minus literal word pairs, and conventional metaphoric minus literal word pairs).

**Fig 10:**
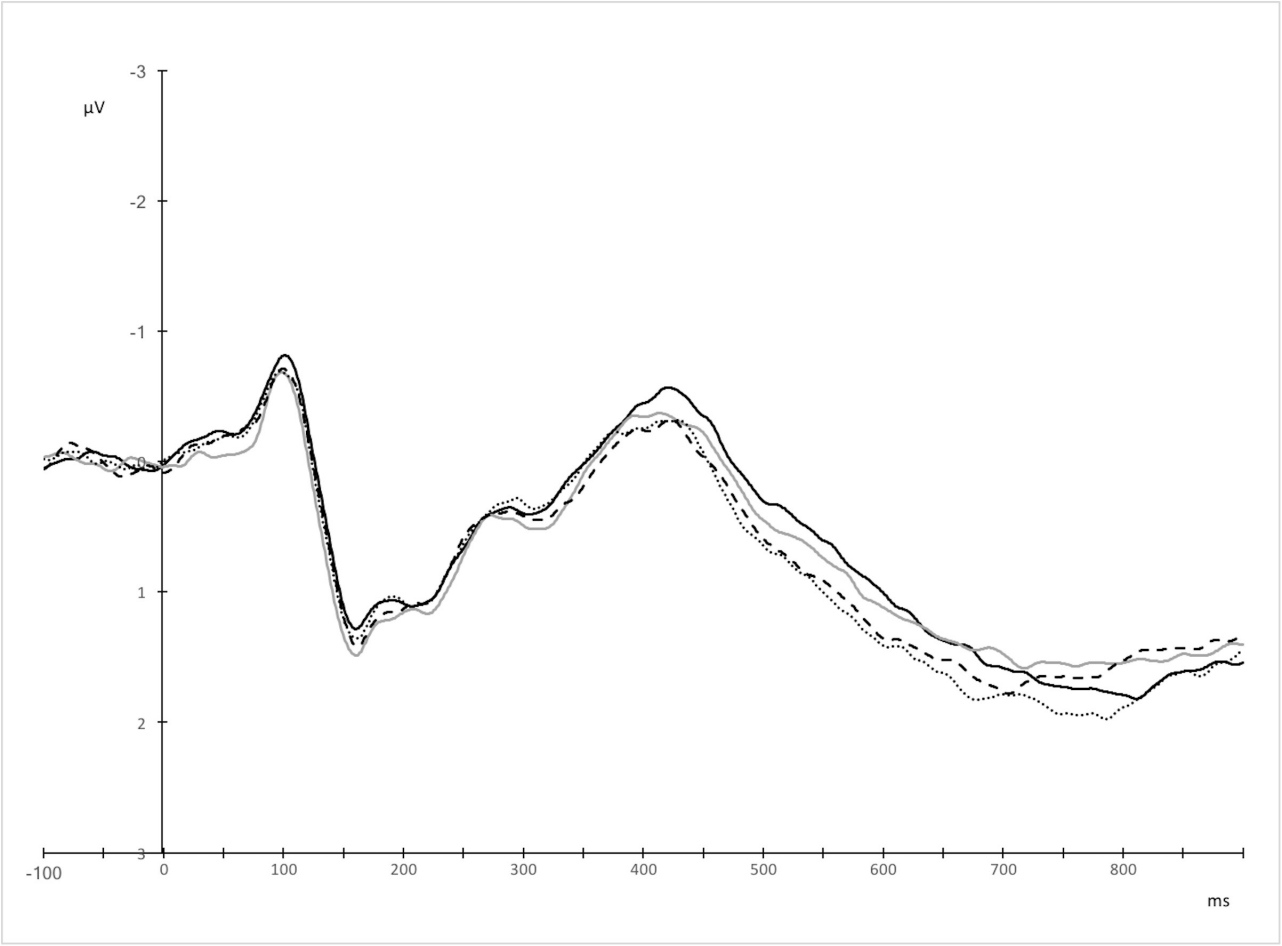
Grand averages for anomalous (black solid line), novel metaphoric (grey solid line), conventional metaphoric (black dashed line), and literal word dyads (black dotted line) over midline and right electrode positions.

Within the 400–500 ms time window, an interaction between utterance type and laterality was found, *F*(12, 264) = 2.95, *p* = .036, ε = .266, η_p_^2^ = .118. To deconstruct the interaction, we computed averaged amplitudes for all left (left and left-medial), all midline, and all right (right and right-medial) electrode positions. For each of these levels of the left-right axis, a repeated measures *ANOVA* was performed on mean amplitudes for the 4 utterance types (novel metaphors vs. conventional metaphors vs. literal utterances vs. anomalous utterances). The results showed a main effect of utterance type over midline, *F*(3, 66) = 4.35, *p* = .007, η_p_^2^ = .165, and right electrode positions, *F*(3, 66) = 4.08, *p* = .01, η_p_^2^ = .156 (Table 10, Fig 9, Fig 10), but not over left electrodes (*p* > .05). Pairwise comparisons showed that anomalous word pairs evoked more pronounced N400 amplitudes relative to literal utterances over midline electrodes (*p* = .014), and relative to conventional metaphors over both midline (*p* = .045) and right electrode positions (*p* = .043). Furthermore, the difference between novel metaphoric and anomalous word dyads was marginally significant over midline electrodes (*p* = .078). A linear effect was additionally observed with maximal amplitudes for anomalous, followed by novel metaphoric, conventional metaphoric, and lowest amplitudes for literal word pairs over midline, *F*(1, 22) = 13.12, *p* = .002, η_p_^2^ = .374, and right electrode positions, *F*(1, 22) = 7.80, *p* = .011, η_p_^2^ = .262, but not over left electrodes (*p* > .05).

**Table 10:** Mean amplitudes for literal (LIT), conventional metaphoric (CM), novel metaphoric (NM), and anomalous (ANO) word pairs over left, midline, and right electrode positions within the 400–500 ms time window.

Electrode position	Mean amplitude for literal utterances	Mean amplitude for conventional metaphors	Mean amplitude for novel metaphors	Mean amplitude for anomalous utterances	Pairwise comparisons
Left	*M* = .655, *SE* = .181	*M* = .625, *SE* = .181	*M* = .585, *SE* = .145	*M* = .669, *SE* = .153	n/a
Midline	*M* = -.012, *SE* = .251	*M* = -.061, *SE* = .261	*M* = -.188, *SE* = .220	*M* = -.349, *SE* = .281	LIT vs. CM (*p* = 1.0); LIT vs. NM (*p* = .970); LIT vs. ANO (*p* = .014); CM vs. NM (*p* = .711); CM vs ANO (*p* = .045); NM vs. ANO (*p* = .078)
Right	*M* = .061, *SE* = .188	*M* = .061, *SE* = .209	*M* = -.051, *SE* = .189	*M* = -.202, *SE* = .233	LIT vs. CM (*p* = 1.0); LIT vs. NM (*p* = .1.0); LIT vs. ANO (*p* = .121); CM vs. NM (*p* = .473); CM vs. ANO (*p* = .043); NM vs. ANO (*p* = .705)

#### 500–800 ms, late positivity

**Fig 11:**
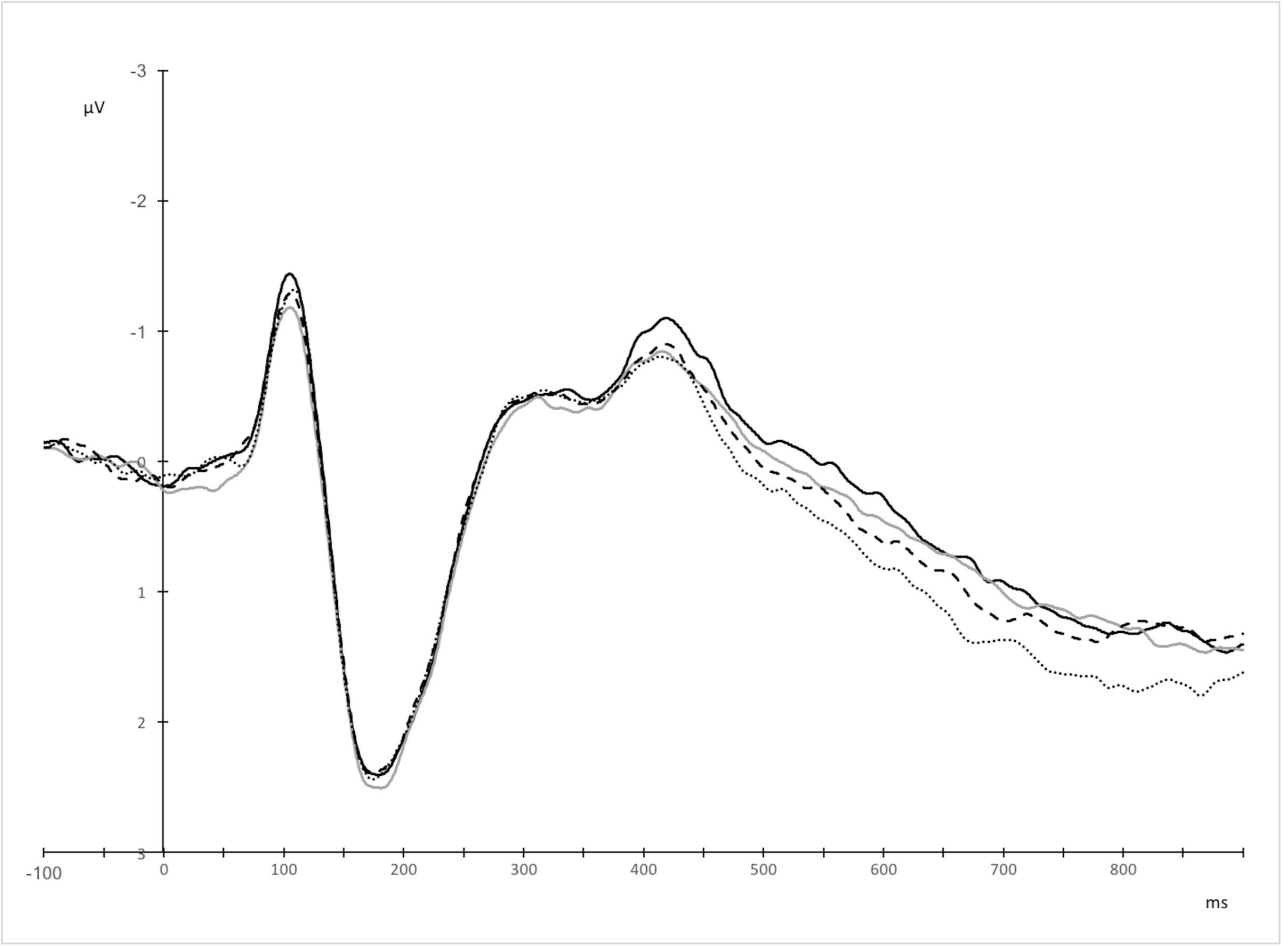
Grand averages for anomalous (black solid line), novel metaphoric (grey solid line), conventional metaphoric (black dashed line), and literal word dyads (black dotted line) over fronto-central and central electrodes.

The analysis of the 500–800 ms time window showed an interaction between anterior-posterior electrode site and utterance type, *F*(9, 198) = 6.87, *p* = .001, ε = .314, η_p_^2^ = .238. To deconstruct the interaction, we computed averaged amplitudes separately for all fronto-central, central, centro-parietal, and parietal electrode positions per each utterance type. For each of these levels of the anterior-posterior axis, a repeated measures *ANOVA* was performed on mean amplitudes for the 4 utterance types (novel metaphors vs. conventional metaphors vs. literal utterances vs. anomalous utterances). The findings showed effects of utterance type over fronto-central, *F*(3, 66) = 12.28, *p* < .001, η_p_^2^ = .358, and central electrode positions, *F*(3, 66) = 8.67, *p* < .001, η_p_^2^ = .283 (Table 11, Fig 11), but not over centro-parietal and parietal electrodes (*p*s > .05). Pairwise comparisons showed that over fronto-central electrodes, literal and conventional metaphoric word dyads evoked larger LPC amplitudes than novel metaphors *(p <* .001, *p* = .002, respectively). Similarly, literal and conventional metaphoric utterances evoked increased positivity relative to anomalous word pairs (*p* = .002, *p* = .03, respectively). Over central electrodes, literal utterances elicited larger LPC amplitudes compared to novel metaphoric and anomalous utterances (*p* = .001, *p* = .002, respectively); and conventional metaphors evoked increased positivity compared to novel metaphoric word dyads, *p* = .031. A linear effect was additionally observed over both fronto-central, *F*(1, 22) = 23.13, *p* < .001, η_p_^2^ = .513, and central electrodes, *F*(1, 22) = 22.55, *p* < .001, η_p_^2^ = .506, with the most pronounced LPC response evoked by literal, followed by conventional metaphoric, novel metaphoric, and finally anomalous word dyads.

**Table 11:** Mean amplitudes for literal (LIT), conventional metaphoric (CM), novel metaphoric (NM), and anomalous (ANO) word pairs over fronto-central, central, centro-parietal, and parietal electrode positions within the 500–800 ms time window.

Electrode position	Mean amplitude for literal utterances	Mean amplitude for conventional metaphors	Mean amplitude for novel metaphors	Mean amplitude for anomalous utterances	Pairwise comparisons
Fronto-central	*M* = .771, *SE* = .250	*M* = .628, *SE* = .235	*M* = .332, *SE* = .264	*M* = .213, *SE* = .280	LIT vs. CM (*p* = .586); LIT vs. NM (*p* < .001); LIT vs. ANO (*p* = .002); CM vs. NM (*p* = .002); CM vs ANO (*p* = .03); NM vs. ANO (*p* = 1.0)
Central	*M* = 1.356, *SE* = .205	*M* = 1.214, *SE* = .203	*M* = 1.031, *SE* = .206	*M* = 1.022, *SE* = .237	LIT vs. CM (*p* = .499); LIT vs. NM (*p* = .001); LIT vs. ANO (*p* = .002); CM vs. NM (*p* = .031); CM vs ANO (*p* = .301); NM vs. ANO (*p* = 1.0)
Centro-parietal	*M* = 2.146, *SE* = .239	*M* = 2.030, *SE* = .214	*M* = 1.881, *SE* = .204	*M* = 2.044, *SE* = .259	n/a
Parietal	*M* = 2.195, *SE* = .260	*M* = 2.158, *SE* = .227	*M* = 2.088, *SE* = .219	*M* = 2.372, *SE* = .254	n/a

Additionally, an interaction was found between language and utterance type, *F*(3, 66) = 4.32, *p* = .008, η_p_^2^ = .164. Follow up analyses were carried out for each language separately. A repeated measures *ANOVA* performed on mean amplitudes for Polish utterances revealed a main effect of utterance type, *F*(3, 66) = 4.65, *p* = .013, ε = .711, η_p_^2^ = .175. Pairwise comparisons further showed that Polish novel metaphors evoked a reduced LPC response compared to conventional metaphors, *p* = .002, and literal word pairs, *p* = .021. No statistically significant differences were found between literal and conventional metaphoric, between anomalous and novel metaphoric, between anomalous and literal, and between anomalous and conventional metaphoric word dyads, *p*s > .05. Additionally, a linear effect across the utterance types was found, *F*(1, 22) = 4.53, *p* = .045, η_p_^2^ = .171, with maximal amplitudes evoked by conventional metaphoric, followed by literal, anomalous, and novel metaphoric word pairs.

**Fig 12:**
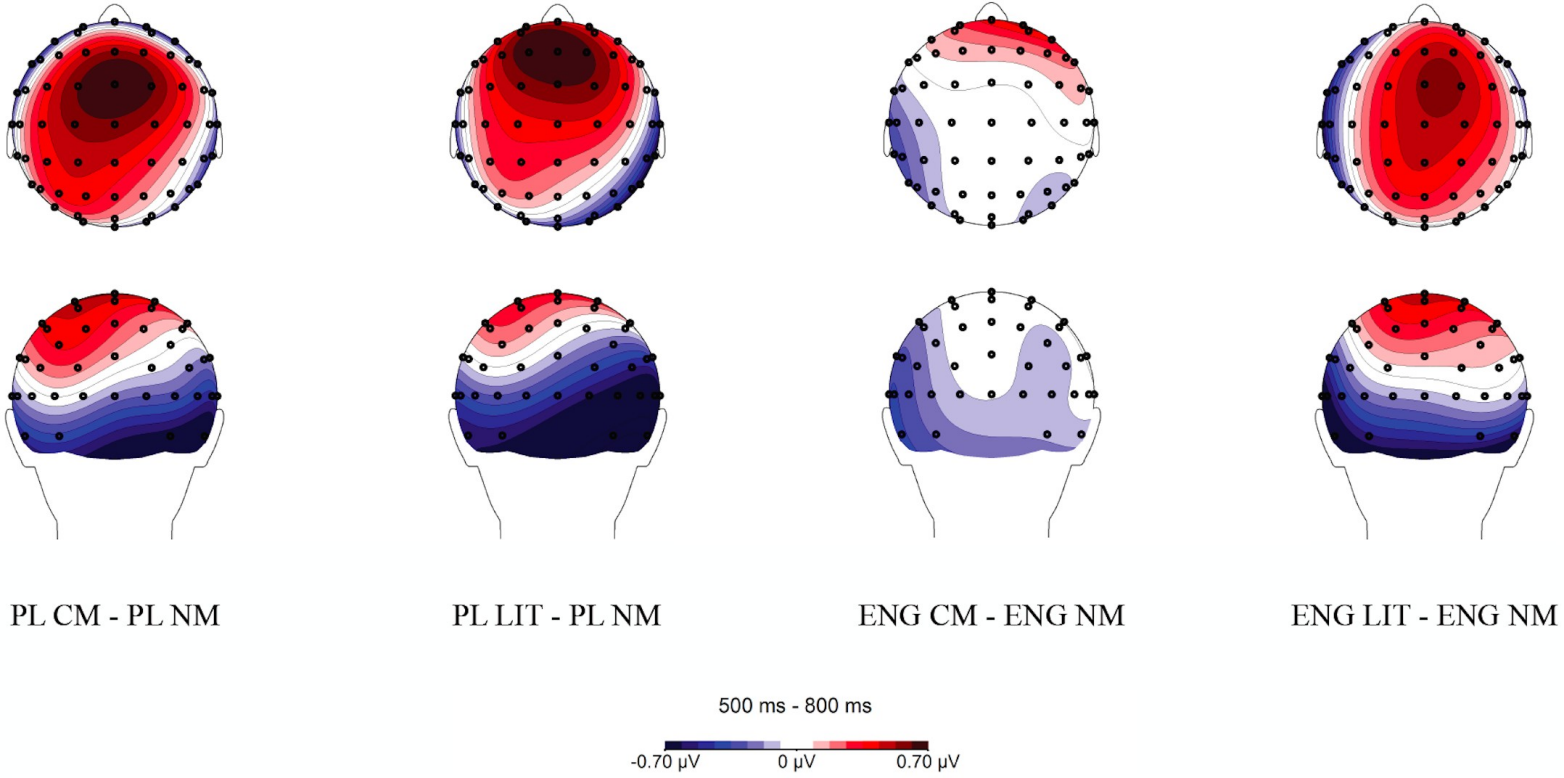
Topographic distribution of novel metaphoric (NM), conventional metaphoric (CM), literal (LIT), and anomalous (ANO) word dyads in Polish (PL) and in English (ENG) within the 500–800 ms time window. Voltage maps were obtained for the averaged value of difference waves (Polish conventional metaphoric minus Polish novel metaphoric word pairs, Polish literal minus Polish novel metaphoric word pairs, English conventional metaphoric minus English novel metaphoric word pairs, and English literal minus English novel metaphoric word pairs).

Similarly, a repeated measures *ANOVA* performed on mean amplitudes for English utterances revealed a main effect of utterance type, *F*(3, 66) = 5.73, *p* = .002, η_p_^2^ = .207. Pairwise comparisons further showed that English literal utterances elicited more positive amplitudes than conventional metaphoric meanings, *p* = .019, and than anomalous word dyads, *p* = .033. No statistically significant differences were found between conventional metaphoric and novel metaphoric, between conventional metaphoric and anomalous, between novel metaphoric and anomalous, and between novel metaphoric and literal word dyads, *p*s > .05. Additionally, a linear effect across the utterance types was found, *F*(3, 66) = 5.44, *p* = .013, η_p_^2^ = .251, with maximal amplitudes elicited by literal, followed by anomalous, novel metaphoric, and conventional metaphoric word dyads (Fig 12). Mean amplitudes for each utterance type in Polish and English are reported in Table 12.

**Table 12:** Mean amplitudes for literal (LIT), conventional metaphoric (CM), novel metaphoric (NM), and anomalous word pairs (ANO) in Polish and English within the 500–800 ms time window.

Language	Mean amplitude for literal utterances	Mean amplitude for conventional metaphors	Mean amplitude for novel metaphors	Mean amplitude for anomalous utterances	Pairwise comparisons
Polish	*M* = 1.639, *SE* = .189	*M* = 1.762, *SE* = .187	*M* = 1.378, *SE* = .165	*M* = 1.503, *SE* = .230	LIT vs. CM (*p* = 1.0); LIT vs. NM (*p* = .021); LIT vs. ANO (*p* = 1.0); CM vs. NM (*p* = .002); CM vs ANO (*p* = .577); NM vs. ANO (*p* = 1.0)
English	*M* = 1.595, *SE* = .204	*M* = 1.253, *SE* = .186	*M* = 1.288, *SE* = .181	*M* = 1.323, *SE* = .217	LIT vs. CM (*p* = .019); LIT vs. NM (*p* = .10); LIT vs. ANO (*p* = .033); CM vs. NM (*p* = 1.0); CM vs ANO (*p* = 1.0); NM vs. ANO (*p* = 1.0)

**Fig 13:**
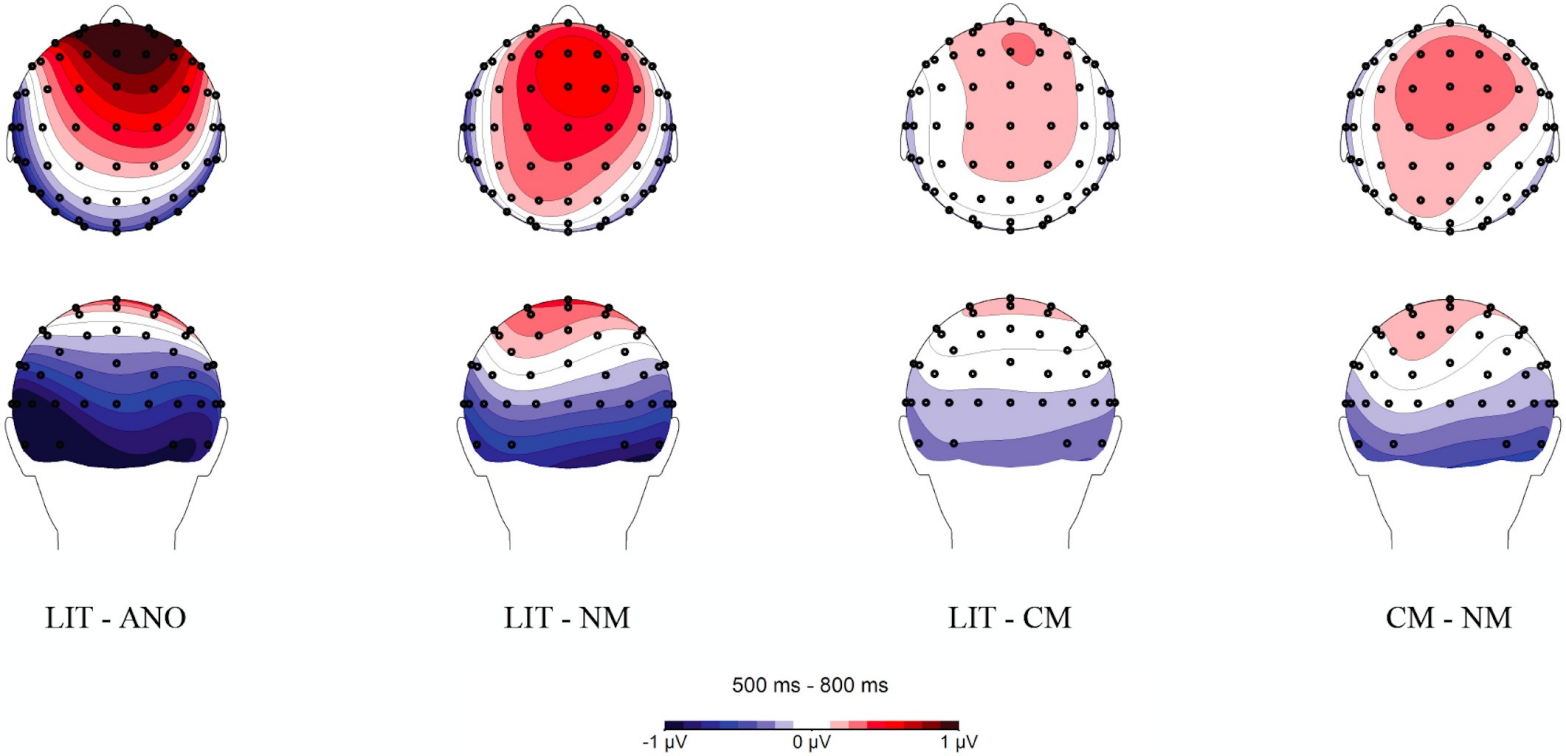
Topographic distribution of novel metaphoric (NM), conventional metaphoric (CM), literal (LIT), and anomalous (ANO) word dyads within the 500–800 ms time window. Voltage maps were obtained for the averaged value of difference waves (literal minus anomalous word pairs, literal minus novel metaphoric word pairs, literal minus conventional metaphoric word pairs, and conventional metaphoric minus novel metaphoric word pairs).

In addition to the interactions, a main effect of utterance type was observed, *F*(3, 66) = 5.89, *p* = .001, η_p_^2^ = .211. Pairwise comparisons showed that literal utterances evoked more positive LPC amplitudes than novel metaphoric, *p* = .003, and conventional metaphoric elicited a more positive LPC response relative to novel metaphoric word dyads, *p* = .015. The difference between literal and anomalous word dyads was marginally significant, *p* = .066. In addition to the main effect, a linear effect for the utterance type was observed, with maximal amplitudes evoked by literal utterances, followed by conventional metaphors, followed by anomalous word pairs, and lowest amplitudes for novel metaphors, *F*(1, 22) = 10.56, *p* = .004, η_p_^2^ = .324 (Table 13, Fig 13).

**Table 13:** Mean amplitudes for literal (LIT), conventional metaphoric (CM), novel metaphoric (NM), and anomalous (ANO) word pairs within the 500–800 ms time window.

Mean amplitude for literal utterances	Mean amplitude for conventional metaphors	Mean amplitude for novel metaphors	Mean amplitude for anomalous utterances	Pairwise comparisons
*M* = 1.617, *SE* = .169	*M* = 1.508, *SE* = .153	*M* = 1.333, *SE* = .151	*M* = 1.413, *SE* = .197	LIT vs. CM (*p* = .823); LIT vs. NM (*p* = .003); LIT vs. ANO (*p* = .066); CM vs. NM (*p* = .015); CM vs ANO (*p* = 1.0); NM vs. ANO (*p* = 1.0)

## Discussion

The present experiment aimed at examining electrophysiological correlates of metaphoric language comprehension in the context of bilingualism, which has thus far been an under-investigated research area. The study addressed the question of whether similar N400 and LPC responses would be evoked by L1 and L2 metaphoric utterances in late proficient unbalanced bilingual speakers. Additionally, the aim of the experiment was to examine differences in cognitive mechanisms when processing metaphors with a different degree of conventionality (i.e., novel and conventional metaphors).

### Behavioral findings

Behavioral analyses revealed an interaction between language and utterance type. While novel metaphors were similarly difficult in both the native and non-native tongue, as indicated by no between-language differences in response times and accuracy rates, conventional metaphors yielded both lower accuracy rates and longer reaction times in English (L2) than in Polish (L1). This finding might indicate an increased difficulty when processing conventional metaphoric meanings in the non-native language compared to the native tongue. Additionally, a main effect of utterance type found in both reaction time and accuracy rate analyses seems to suggest that metaphoric language comprehension is modulated by the degree of conventionality, with novel metaphors eliciting overall longer response times and lower accuracy rates than conventional metaphors. This finding provides support for the Career of Metaphor Model [5], showing that processes involved in meaning construction (novel meanings) are more time-consuming than those engaged in meaning retrieval (conventionalized meanings).

It might be argued that the observed effect may be due to lower frequency of conventionalized metaphors in L2 than in L1, as a result of which they were perceived as less meaningful and evoked longer RTs. However, a strong positive correlation between accuracy rates from the EEG experiment and meaningfulness ratings from the normative studies in both Polish and English was found. This finding is of special importance with respect to conventional metaphors, where the correlation between meaningfulness ratings and accuracy rates was similarly strong in Polish and English. Since the meaningfulness of English conventional metaphors was assessed by English native speakers in the normative study, and by L2 English learners in the EEG experiment, the observed correlation indicates that both English native speakers and English L2 learners made similar decisions regarding the meaningfulness of conventional metaphors.

In addition to the interaction, behavioral findings also showed language-specific differences that were irrespective of utterance type, with lower accuracy rates as well as longer response times elicited by English than Polish utterances, thus indicating that semantic processing might be more cognitively taxing in L2 than in L1. Such an effect may stem from the fact that participants were L1-dominant, and used their native tongue more frequently than their second language. Consequently, the subjective frequency of the non-dominant lexical items is likely to be lower, resulting in slower and less automatic cognitive mechanisms engaged in L2 processing [49].

### Event-related potentials

The first aim of the current study was to examine brain responses to novel metaphoric, conventional metaphoric, literal, and anomalous word pairs in Polish (L1) and English (L2), as indexed by the N400 response. In line with what was hypothesized, we observed general between-language differences, with English utterances eliciting smaller N400 amplitudes than Polish word dyads over central, centro-parietal, and parietal electrode positions, with a slight right hemisphere bias. This effect was observed within the early N400 time window (300–400 ms), and was independent of utterance type. An attenuated N400 response to L2 relative to L1 stimuli has previously been observed in research on bilingual semantic processing [50–54], and has been linked to weaker semantic interconnectivity for L2 relative to L1 words within the memory system [51]. As proposed by Midgley et al. [51], the N400 language nativeness effect (larger N400 amplitudes for L1 than L2 items) might be explained by larger interconnectivity for L1 items within the semantic network. Such larger interconnectivity might evoke increased activity in long-term memory, which seems in line with the current views on the functional role of the N400 response, according to which N400 amplitudes reflect effort related to information retrieval from long-term memory [23,25,57].

In addition, the comparison of the N400 peak latency in L1 and L2 within the 300–500 ms time window showed a 20 ms delay in response to English relative to Polish word dyads, which is in accordance with previous research on the N400 in bilingual semantic processing [44–47]. The obtained findings support the temporal delay assumption postulated by the BIA+ model [49], according to which the activation of semantic representations is delayed in the non-native tongue due to lower subjective frequency of L2 items in L2 non-dominant bilingual speakers. This consequently results in less automatic operations engaged in lexico-semantic access when processing the non-dominant language, owing to its lower resting level activation. Since subjective frequency was not directly measured in the present study, future research could further elucidate the influence of subjective frequency on the N400 amplitudes.

Another factor that might have influenced the obtained findings is the age of L2 acquisition, as our participants acquired their L2 after the age of nine. This would indicate that brain mechanisms engaged when processing the non-native tongue may be less automatic when the foreign language was acquired later than the native tongue. Such an interpretation is in line with Weber-Fox and Neville [47], who highlighted the role of age of L2 acquisition (AoA) in the N400 response by reporting modulations in the N400 effect in participants who acquired their L2 after the age of ten. However, Midgley et al. [51] found no differences between the N400 waveforms elicited by L2 items acquired before and after the age of eight, therefore questioning the influence of late AoA on the N400 amplitudes.

The second finding within the N400 time frame we observed was the graded effect of utterance type over midline and right electrode positions in the late N400 time frame (400–500 ms), which was independent of language. Smallest N400 amplitudes were evoked by literal, followed by conventional metaphoric, novel metaphoric, and finally anomalous word dyads. Importantly, the lack of interaction between language and utterance type suggests that language did not influence cognitive mechanisms involved in lexico-semantic processes during nonliteral meaning comprehension, which are indexed by the N400 response. Such a linear effect across the utterance types is in line with previous monolingual research on metaphor comprehension, and indicates an increasing trend of resource intensity of mappings between concepts from literal, to conventional metaphoric, novel metaphoric, and anomalous utterances [18,29]. The findings provide support for the Career of Metaphor Model [5], which postulates that mechanisms engaged in metaphor comprehension are modulated by metaphor conventionality. Since novel metaphors require meaning construction, they involve lexico-semantic processes that are more taxing than those involved in meaning retrieval during conventional meaning comprehension. In addition, the fact that conventional metaphors elicited a larger N400 response than literal meanings might indicate that conventional metaphoric utterances, in spite of their high familiarity and frequency of use, require more resource intensive mappings between concepts than literal utterances [17,31].

Our third aim was to examine whether cognitive mechanisms involved in meaning integration, as indexed by the LPC response, are modulated by language nativeness. Within the LPC time frame (500–800 ms), we observed between-language differences in response to individual utterance types, with a smaller LPC response evoked by novel than conventional metaphors and literal utterances, yet only in Polish. In English, on the other hand, reduced amplitudes within the LPC time window were observed for novel as well as conventional metaphoric word dyads relative to literal utterances. This effect might reflect a negative component overlapping with the LPC time window for novel metaphoric word pairs in L1, and for novel and conventional word dyads in L2. Additionally, within the LPC time frame, we also observed differences between utterance types over the fronto-central and central sites, which were irrespective of language. Although we expected a more pronounced LPC response to novel metaphoric and anomalous word dyads, they instead evoked smaller LPC amplitudes than conventional metaphoric and literal word pairs.

Decreased LPC amplitudes for novel metaphoric word dyads could be interpreted in light of findings on sustained negativity, which has been linked to the processes of integration of conceptually difficult meanings, as well as operations involved in reprocessing after an initial failure in meaning interpretation [43,58], or additional working memory load during complex stimuli processing [58–61]. In research on metaphors, smaller LPC amplitudes for novel metaphoric than literal utterances have previously been observed [18], which was interpreted as indicative of continued effort related to information retrieval or access to the non-literal route when interpreting novel metaphors. Similar findings were also observed by Rutter and colleagues [62], who found sustained negativity in response to novel metaphors and anomalous sentences in a delayed response procedure, and interpreted it as continuation of the N400 effect, reflecting the ongoing difficulty of meaning integration. While in the current study reduced LPC amplitudes were maximal over fronto-central and central electrodes, in the study reported by Rutter and colleagues the effect was observed over central, centro-parietal, and parietal electrode sites. This dissimilarity might, however, stem from differences in experimental procedures between the two studies.

Reduced LPC amplitudes for novel metaphors observed in the current study might be therefore interpreted as continued effort related to information retrieval or access to the non-literal route during unfamiliar meaning comprehension in both languages [18,43,58,59]. In the non-native language, however, LPC amplitudes for conventional and novel metaphors converged, suggesting increased cognitive effort to integrate the meaning of not only novel, but also conventional metaphoric word pairs. This novel result might indicate decreased sensitivity to the levels of conventionality of metaphoric meanings in late proficient unbalanced bilingual speakers in later stages of metaphoric language processing (500–800 ms). Importantly, this effect is in line with the behavioral results observed in the present study, which showed longer response latencies as well as lower accuracy rates for English than Polish conventional metaphors.

Finally, although the aims of the current paper did not include any specific predictions regarding the P200 component, visual inspection revealed diverging waveforms for L1 and L2 as early as 150 ms after stimulus onset. More robust P200 amplitudes were observed for English than Polish word dyads. The P200 response has been found to be modulated by a number of cognitive tasks and higher-order perceptual processing, including attention, short-term memory, early item encoding, as well as expectancy for a given word [63–68]. Though the exact functional role of the P200 in language processing research is still under discussion, some evidence suggests that the component marks cognitive mechanisms related to early lexical access [69]. Namely, a number of studies have reported a more pronounced P200 response to low than high frequency words, indicating increased processing cost for such items [70–72].

In light of the present findings, a more robust P200 response to L2 than L1 stimuli observed in the current study might stem from a lower subjective frequency of English than Polish stimuli. Due to the fact that participants were Polish-dominant bilinguals, the subjective frequency of lexical items belonging to their non-dominant language (i.e., English) might have been lower, resulting in a more pronounced P200 effect. The findings might therefore suggest a priority of L1 words, whose lexical access may be completed faster, while L2 items are still being processed [72].

As shown by the present findings, between-language differences in response to metaphors were not observed until the LPC time window, in which conventional metaphoric word dyads evoked reduced LPC amplitudes only in L2, while for novel metaphors, LPC amplitudes were attenuated in both languages. Such an effect points to continued effort in information retrieval or access to the non-literal route during novel metaphor comprehension in L1, and during both novel and conventional metaphor comprehension in L2. Also, a language-independent graded effect was found within the late N400 time window, which indicates a more resource intensive lexico-semantic operations engaged in novel compared to conventional metaphor comprehension in both languages. Additionally, language specific differences were found within the P200 time frame, suggesting lower subjective frequency of L2 lexical items, as well as within the early N400 time window, indicating less automatic lexical access, extended lexical search, as well as reduced interconnectivity for L2 words.
